# A Minispidroin Guides the Molecular Design for Cellular Condensation Mechanisms in *S. cerevisiae*

**DOI:** 10.1021/acssynbio.3c00374

**Published:** 2023-09-09

**Authors:** Jianhui Feng, Bartosz Gabryelczyk, Isabell Tunn, Ekaterina Osmekhina, Markus B. Linder

**Affiliations:** Department of Bioproducts and Biosystems, School of Chemical Engineering and Academy of Finland Center of Excellence in Life-Inspired Hybrid Materials (LIBER), Aalto University, Espoo 02150, Finland

**Keywords:** liquid−liquid phase separation, intracellular protein condensates in yeast, spidroin, synthetic condensates, liquid to solid transitions, protein-based materials

## Abstract

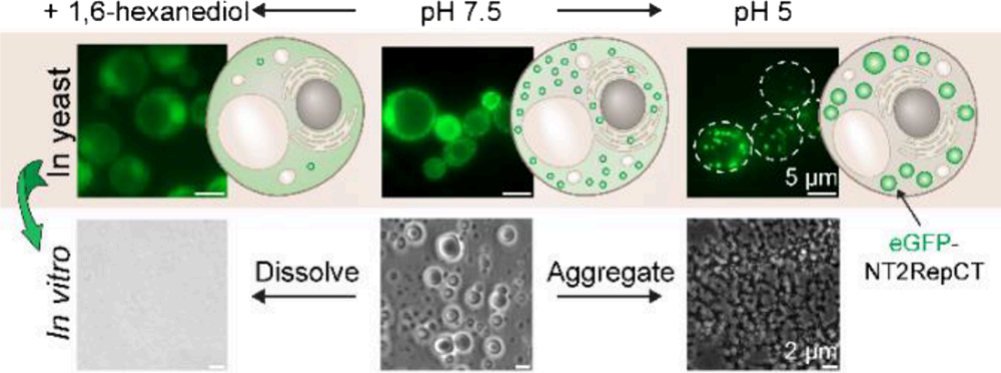

Structural engineering of molecules for condensation is an emerging technique within synthetic biology. Liquid–liquid phase separation of biomolecules leading to condensation is a central step in the assembly of biological materials into their functional forms. Intracellular condensates can also function within cells in a regulatory manner to facilitate reaction pathways and to compartmentalize interactions. We need to develop a strong understanding of how to design molecules for condensates and how their *in vivo–in vitro* properties are related. The spider silk protein NT2RepCT undergoes condensation during its fiber-forming process. Using parallel *in vivo* and *in vitro* characterization, in this study, we mapped the effects of intracellular conditions for NT2RepCT and its several structural variants. We found that intracellular conditions may suppress to some extent condensation whereas molecular crowding affects both condensate properties and their formation. Intracellular characterization of protein condensation allowed experiments on pH effects and solubilization to be performed within yeast cells. The growth of intracellular NT2RepCT condensates was restricted, and Ostwald ripening was not observed in yeast cells, in contrast to earlier observations in *E. coli*. Our results lead the way to using intracellular condensation to screen for properties of molecular assembly. For characterizing different structural variants, intracellular functional characterization can eliminate the need for time-consuming batch purification and *in vitro* condensation. Therefore, we suggest that the *in vivo–in vitro* understanding will become useful in, e.g., high-throughput screening for molecular functions and in strategies for designing tunable intracellular condensates.

## Introduction

Liquid–liquid phase separation (LLPS), also referred to as coacervation or condensation, enables sequestering specific biomacromolecules (e.g., proteins and nucleic acids) into liquid-like condensates that serve as subcellular compartments*–*membraneless organelles.^1,2^ LLPS is increasingly being understood to have a universal and important role in processes by which cells achieve spatial and temporal control of several of their functions.^1−3^ Formation of intracellular liquid-like condensates allows dynamic exchange of components with the surroundings.^2,3^ Moreover, the viscoelastic material properties of condensates can alter from liquid-like to solid-like in response to the changes of the intracellular biochemical environment or external biophysical stimuli.^2,4,5^ These alterations are closely linked to their cellular functions.

In parallel, research in the field of extracellular protein-based biomaterials has revealed that LLPS is a key initial step in their molecular assembly pathways.^6^ Examples are squid beak,^7,8^ elastin fibers,^9^ mussel adhesives,^10,11^ and spider silk.^12,13^ Within the condensates, the conformational entropy of the molecules is reduced, and their concentration is increased.^14^ These effects collectively contribute to the organization of the molecules toward their final material state. Similar to many eukaryotic LLPS proteins (e.g., RNA binding proteins), some biomaterial building blocks are multidomain proteins having both intrinsically disordered regions (IDRs) and folded domains.^15−18^ An example of such multidomain arrangements is the silk-forming proteins, e.g., the spidroins.^19,20^ The presence of IDRs has been found to be the key for LLPS because IDRs offer high conformational flexibility and multivalent interactions, whereas the folded domains connecting IDRs usually mediate LLPS propensity.^21,22^

The increasing understanding of molecular grammars governing and mediating LLPS has incentivized efforts to make new intracellular condensates. Several recent reports described modified proteins used to construct artificial intracellular condensates that resemble naturally occurring condensates in living cells, including building blocks of biomaterials.^23−26^ Studies showed that intracellular condensates formed by resilin-like or spider silk-mimicking proteins can lead to selective colocalization of macromolecules that can be beneficial for affecting reaction rates, creating novel ways of regulation, or improving the solubility of intermediates.^26−28^ Of particular interest is that the viscosity and material properties of synthetic condensates can be tuned for desired applications by, e.g., changing amino acid composition or polypeptide length and modifying or adding IDRs.^24,26^

Microbial production of protein-based materials is attractive within synthetic biology because it is important for reaching sustainability goals and allows tailored functions. However, improving their microbial production is still a major challenge.^29^ Many proteins for biomaterials contain IDRs, and they are highly repetitive, are prone to aggregation, and have high molecular weight.^30,31^ Heterologous expression of these proteins often results in metabolic burden and premature protein aggregation in the host cells.^32^ In addition, we have an incomplete understanding of the molecular mechanism by which material-forming proteins gain their functional interactions and how these are connected to the mechanisms of LLPS.^33−35^ This hinders rational protein design strategies for biological materials and usually translates into laborious and tedious work on the purification of multiple indeterminate protein candidates and characterization under different conditions *in vitro*.

We need an approach to facilitate screening for potential protein variants for functional material production. Inspired by the intracellular phase separation of various material building blocks into condensates, we propose that *in vivo* characterization of condensates can be a useful strategy for protein engineering for biomaterial production. Traditional *in vitro* LLPS protein characterization could be sped up if physical*–*chemical properties such as the tendency to undergo LLPS and the properties of condensates could be assessed already within cells, i.e., using the cells as “living test tubes” for protein condensates. For this, we need to build a fundamental understanding of how characterizations of LLPS *in vitro*–*in vivo* are correlated. This understanding is also important generally for cellular engineering with synthetic condensates.

We approached this general concept by using a model minispidroin NT2RepCT that mimics the overall domain structure of the native spider silk spidroin.^36,37^ NT2RepCT is a triblock protein consisting of an IDR (2Rep) in the center capped by a globular N-terminal domain (NT) and a C-terminal domain (CT).^36−38^ The 2Rep has two repeats of polyalanine (poly-A) stretches alternating with polyglycine (poly-G) blocks.^36^ CT functions as a constitutive homodimer, whereas NT is highly soluble monomer at pH above 6.5 and forms dimers at lower pH.^39,40^ Spider silk formation that occurs at low pH (e.g., pH 5) is a coordinated and organized event. Whereas the dimerization of NT interconnects the spidroins into large networks, CT gets destabilized and conformationally converts into β-sheet structures that trigger a subsequent structural transformation of the central repetitive region into β-sheets.^36,40^ Full-size spidroins with a long IDR generally have a low microbial production yield, but NT2RepCT exhibits high production yield in *E. coli* (20 g/L) and high solubility.^36,37^ Most importantly, NT2RepCT still forms continuous fibers with closely matched mechanical properties compared to the native silk.^37^

Our previous study has shown that NT2RepCT can undergo LLPS in *E. coli* cells.^41^ The *in vivo* LLPS can be important for enhancing its solubility during production. Similarities between properties of *in vivo* and *in vitro* condensates were shown, and it was proposed that the LLPS preassembly of NT2RepCT in *E. coli* cells closely links to its functional fibrillization *in vitro*. We wondered whether LLPS of NT2RepCT can occur and be characterized in eukaryotic systems, e.g., yeast cells, and about the correlations of condensate formation and properties between yeast cells and *in vitro*. The answer could expand our knowledge on the phase behaviors of material proteins in different cellular systems, give more insights into the molecular basis of designing synthetic condensates, and evaluate the feasibility of eukaryotic cell as an additional avenue for *in vivo* analysis of material protein properties. In this study, we first overexpressed NT2RepCT and studied the properties and dynamics of its intracellular structures in *Saccharomyces cerevisiae*. We found that NT2RepCT spontaneously assembled into small granules with “liquid condensate” like properties that became solid-like over time and can undergo further assembly into larger granules upon artificially decreasing cytosolic pH. A similar trend was seen *in vitro* where NT2RepCT underwent low pH induced transitions from liquid condensates to fibrils. Moreover, the capability of undergoing self-assembly and pH-regulated solidification characterized for all truncation variants of NT2RepCT in yeast cells showed high similarity to that *in vitro*. Thus, we established that both acidification and LLPS are essential for fibrillization of NT2RepCT. In the end, we proposed the feasibility of using yeast cells as a living test tube for characterizing condensates, but how the cellular environment affects the condensation of structural proteins needs to be better understood when comparing the *in vivo* condensate analysis to that *in vitro*.

## Results

### During Overexpression in Yeast, NT2RepCT Forms Liquid-like Granular Structures that Become Solid-like Over Time

NT2RepCT was tagged with an enhanced green fluorescent protein (eGFP) at its N-terminus (Figure S1), which allowed visualization by fluorescence microscopy. The eGFP tagged version of NT2RepCT was used throughout this study. It is called NT2RepCT for simplicity. The galactose-inducible promoter (Gal1) was used for tight control of NT2RepCT expression in *S. cerevisiae*.^42^ Gal1 allows heterologous protein expression with a broad range of concentrations depending on the induction time. The tight control of protein expression enables the investigation of the direct influence of protein concentration on its phase behavior by minimizing growth-related perturbations. Expression of NT2RepCT was induced with 2% galactose at the early exponential growth phase, and protein behavior was monitored in the following 16 h.

**Figure 1:**
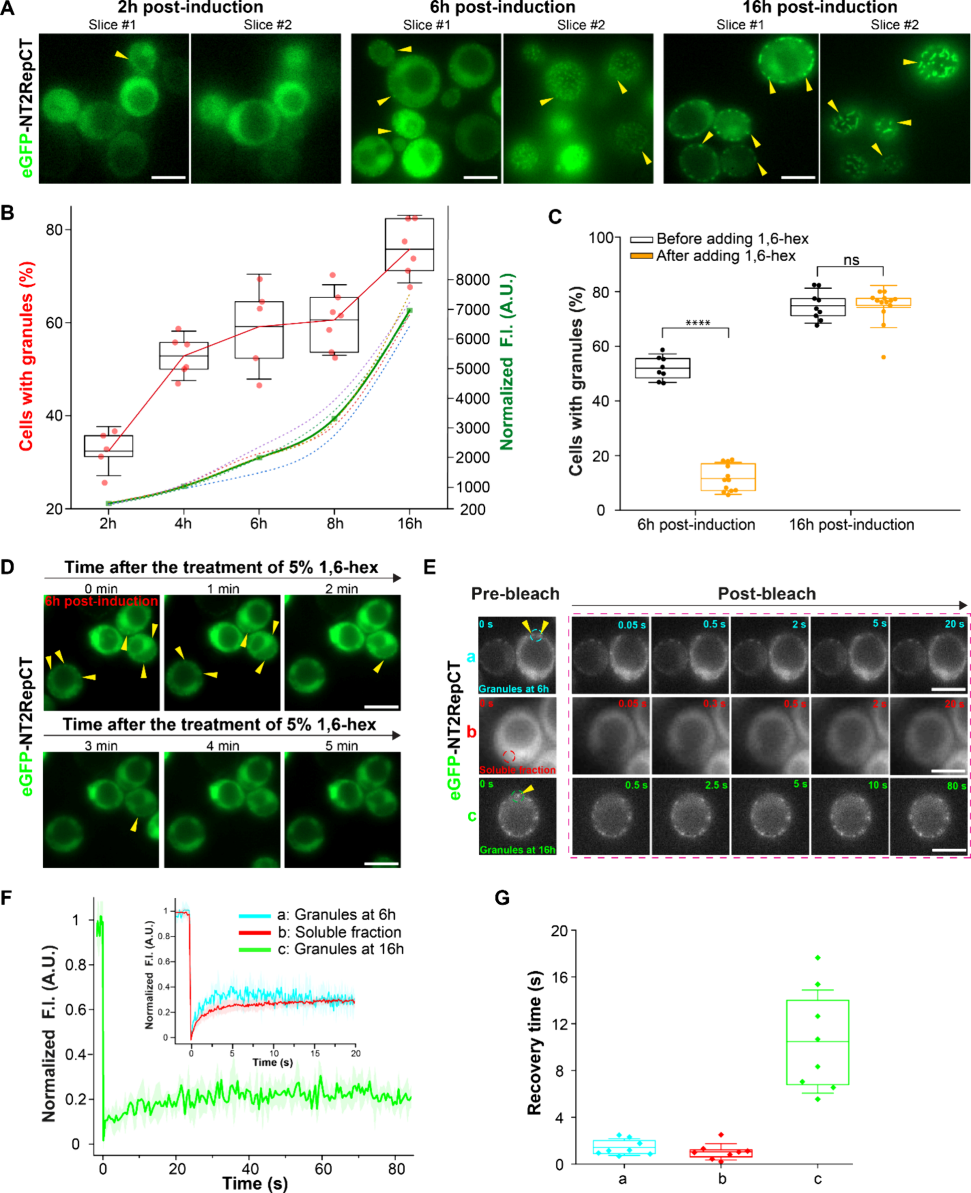
NT2RepCT readily forms 1,6-hex dissolvable granules in yeast cells at the early growing phase that becomes solid-like in aged cells. (A) Fluorescence microscopy images of NT2RepCT expressed in yeast cells at different time points postinduction. Cells were grown in the SC-His media containing 2% of galactose. Slice #1 is a single *z*-plane image taken when the focal plane was around the cell equator, and slice #2 is an image taken when the focal plane was around 0.2 μm above the cell equator. Different settings were used during image processing, so intensities are not directly comparable. (B) Quantification of cells containing NT2RepCT granules and the overall GFP intensity normalized by OD600 as a function of induction time. Red dots represent different biological replicates (at least five biological replicates). Five biological replicates (represented by different colors of dash lines) were conducted to map the trend of overall GFP intensity as a function of induction time. The bold green line represents the average of the overall normalized GFP intensity. (C) Quantification of cells containing NT2RepCT granules in the presence of 5% 1,6-hex after the protein was expressed for 6 and 16 h. Asterisks represent statistical significance between conditions indicated by horizontal bar ends: **** = *p* < 0.0001, *** = *p* < 0.001, ** = *p* < 0.001, * = *p* < 0.05, and ns = not significant (*p* > 0.05). (D) Time-lapse images of yeast cells expressing NT2RepCT for 6 h after being treated with 5% 1,6-hex. (E) Fluorescence recovery after photobleaching (FRAP) images of cells expressing NT2RepCT captured at different time points before and after photobleaching. (a) Small granules of NT2RepCT formed after 6 h of induction, (b) soluble fraction of NT2RepCT after 2–4 h of induction, and (c) small granules of NT2RepCT formed after 16 h of induction. Round dash circles roughly represent the photobleaching area of the cells. (F) Recovery of fluorescence of NT2RepCT at different conditions. The bold lines represent the average recovery of fluorescence over time, and the standard deviations of each condition are plotted in the form of a shaded area. (G) Plot of the characteristic time of fluorescence recovery of NT2RepCT at different conditions corresponding to panel F. Red dots represent different samples for measuring (eight replicates for each condition were used for calculation). In all box plots, the mean is shown as a line in the middle or outside of the box, and the box shows lines at 25th and 75th percentiles. Whiskers show the standard deviation values. Yellow arrows indicate NT2RepCT granules within cells. Scale bar in all of the microscopic images is 5 μm.

Two hours after the expression of NT2RepCT, most cells exhibited diffuse fluorescence with a relatively low overall intensity (Figure 1A,B). Some small fluorescent puncta were observed in a few cells (Figure 1A and Figure S2). In the following 2–8 h, the overall fluorescence intensity increased gradually, and the number of puncta per cells noticeably increased (Figure 1A). The portion of cells containing such puncta also increased to around 60% (Figure 1B). The puncta were distributed around the cellular periphery and were morphologically similar to biomolecular condensates in yeast cells, such as stress granules.^15,16^ The more general term granules is used in the following description, although they putatively were considered to be condensates. Considering that the effect of growth-related disturbance on protein behavior is minimal within this induction period (2–8 h), the increasing granule formation hints that NT2RepCT at a low concentration in yeast cells initially exists in a soluble form and that it is prone to associate together into granules as a more condensed form in response to elevated protein concentration. Although increasing protein concentration resulted in more granules present in the cells, they did not grow in size by fusion as expected if they had liquid-like properties.^24,43^ When the induction was extended to 16 h, the number of cells containing granules reached approximately 80% as fluorescence intensity continuously increased (Figure 1A,B). Unexpectedly, at this later stage, apart from granules, we observed some rod-like and elongated structures of NT2RepCT (Figure 1A and Figure S2). Because the yeast’s cytoplasm in the late-growing phase (16 h) might experience alterations in viscosity, pH regulation, and ion homeostatics,^15,44^ it was uncertain to what extent these rod-like structures were caused by increased protein concentration or an environmental change of cytoplasm.

We next investigated if the structures of NT2RepCT formed in cells at 6 and 16 h postinduction differed in their physical properties. First, we treated the cells with 1,6-hexanediol (1,6-hex), which is commonly used to probe material properties of condensates.^45^ Most granules that had formed within 6 h postinduction dissolved within 5 min after the treatment of 5% 1,6-hex (Figure 1C,D). Almost none of the granules at 16 h postinduction dissolved with 5% or even 10% 1,6-hex (Figure S3A), similar to that of the control samples without adding 1,6-hex (Figure S3B), suggesting looser and more dynamic interactions within the NT2RepCT granules formed at 6 h than those at 16 h. Fluorescence recovery after photobleaching (FRAP) was used to further probe the fluidity of granules.^46^ Because of the small size of these two different granules, it was not possible to achieve a partial photobleaching of them to investigate their internal dynamics, so we were restricted to photobleaching the whole granules. We noted that granules formed at 6 h did regain around 30% of their prebleached fluorescence intensity within 1 to 2 s (Figure 1E–G). The recovery time and magnitude were both similar to those of the diffuse cytosolic fraction of NT2RepCT 2–4 h postinduction and those of the reference soluble protein eGFP in the cytosol (Figure 1E–G and Figure S4). However, the granules formed at 16 h after induction, including dot-like and rod-like structures, exhibited a slower recovery of around 20% with an average recovery time of 9 s (Figure 1E–G). Although both intracellular structures—formed at 6 and 16 h—of NT2RepCT showed a certain level of fluorescence recovery after photobleaching, we did not observe the reappearance of respective intracellular structures on the bleached area even after 2 min. It was assumed the recovery could also result from the diffusion of the soluble fraction of NT2RepCT to the bleached area. Therefore, our FRAP data did not directly suggest liquid properties of intracellular structures of NT2RepCT but instead showed a difference in diffusivity of NT2RepCT molecules between 6 and 16 h postinduction.

To address the possibility that 1,6-hex resistant granules at 16 h postinduction are misfolded protein aggregates that might affect cell physiology, we used a reporter system that was designed to identify whether imported proteins form cytosolic aggregates in yeast based on the cellular unfolded protein response (UPR).^47^ In the reporter system, cells produce GFP fluorescence in the presence of cytosolic aggregates that trigger UPR.^47^ For these experiments, we tagged NT2RepCT with a red fluorescent protein (RFP) at its N-terminal. RFP-NT2RepCT was expressed in the reporter strain and formed small granules consistent with that of the eGFP tagged variant (Figure S5A). The control aggregated protein RFP-LipPks formed large and irregular-shaped granules and stimulated threefold enhancement of GFP fluorescence compared to the control soluble protein RFP-MBP (maltose binding protein) (Figure S5). Cells expressing RFP-NT2RepCT gave rise to an equivalently low fold-change of GFP fluorescence as a control reporter strain without heterologous protein expression and the strain expressing RFP-MBP (Figure S5). This indicates that overexpression of NT2RepCT for 16 h did not upregulate the cellular UPR compared to misfolded LipPks and that no evidence for NT2RepCT forming misfolded protein aggregates in cells was found.

### NT2RepCT Rapidly Forms Larger and More Protein-Enriched Assemblies upon Decreasing Cytosolic pH

**Figure 2:**
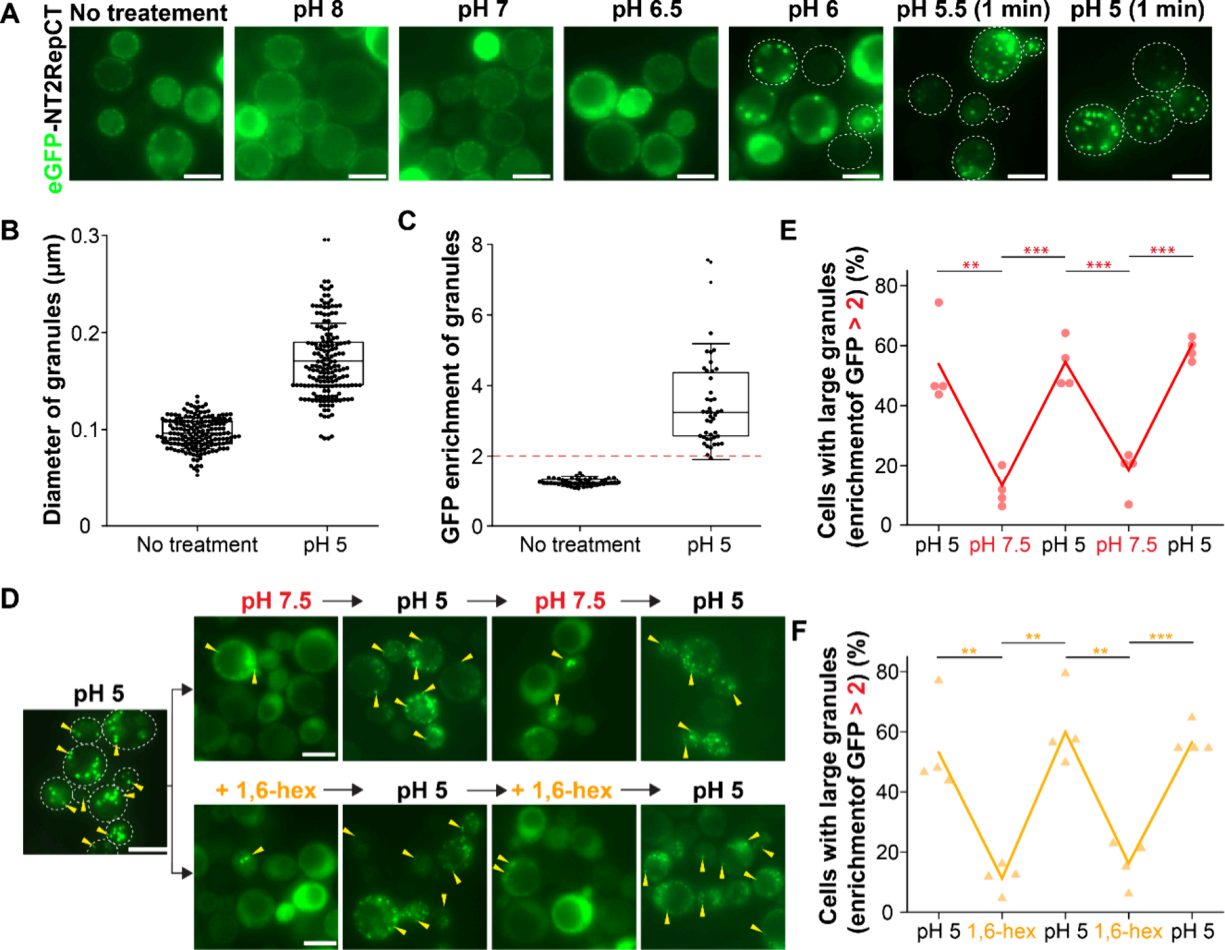
NT2RepCT at lower cytosolic pH assembles into larger and more protein-enriched granules that are pH reversible and 1,6-hex dissolvable. (A) Fluorescence microscopy images of yeast cells expressing NT2RepCT incubated in DNP-containing buffers at different pH values. The white dashed circles roughly represent the cell border. (B) Diameter of granules before and after the pH treatment. Each dot represents the diameter of an individual granules in different cells. (C) Enrichment of eGFP ([fluorescence intensity inside clusters]/[average fluorescence intensity of cytoplasm excluding clusters]) before and after the pH treatment. Each dot represents the GFP enrichment of individual granules in different cells. The red dash line depicts the threshold GFP enrichment ratio of 2, which the NT2RepCT granules in the nontreated cells do not exceed. (D) Fluorescence microscopy images of yeast cells during the experiment of the investigation of pH reversibility and 1,6-hex dissolvability of NT2RepCT. Yellow arrows depict the NT2RepCT granules. (E) Quantification of the cells containing granules (with GFP enrichment ratio >2) in repeated treatment cycles of pH 5 to 7.5. Red dots represent different biological replicates. (F) Quantification of the cells containing cellular granules (with GFP enrichment ratio >2) in repeated cycles of 1,6-hex treatment. Orange labels represent different biological replicates. Asterisks represent statistical significance between conditions indicated by horizontal bar ends: **** = *p* < 0.0001, *** = *p* < 0.001, ** = *p* < 0.001, * = *p* < 0.05, and ns = not significant (*p* > 0.05). In all box plots, the mean is shown as a line in the middle of the box, and the box shows lines at 25th and 75th percentiles. Whiskers show standard deviation values. Scale bar in all microscopic images is 5 μm.

In the spider silk gland, the assembly of spidroins into fibrillar structures is triggered by exposure to low pH at around 5 to 5.5.^36,37,48^ Therefore, we tested the response of intracellular NT2RepCT to different pH values. 2,4-Dinitrophenol (DNP) (2 mM) was used together with buffers to change intracellular pH. Because DNP can shuttle protons across the plasma membrane, the cytosolic pH and the external pH are equilibrated.^49^ The cytosolic pH of exponentially growing yeast cells is around pH 7.5,^49^ which is expected to be the cytosolic pH of cells expressing proteins for 2–6 h. After adding DNP-containing buffers with pH above 6.5 to cells expressing NT2RepCT for 6 h, the small and round granules of NT2RepCT showed no significant difference in appearance compared to before the treatment (Figure 2A). In a DNP-containing buffer with a pH of 6, we observed the formation of larger and brighter granules (Figure 2A). Upon further decreasing pH to 5 and 5.5, an increasing number of large granules were seen (Figure 2A,B). The formation of large granules occurred within 1 min after the addition of DNP-containing buffer at pH 5 to the cells (Figure 2A and Figure S6). The fluorescence intensity within the large granules formed at pH 5 could reach around 7 times higher than the average cytoplasmic fluorescence (with an average GFP enrichment ≈3.8) (Figure 2C). The small granules formed spontaneously at physiological pH had a much lower GFP enrichment ratio of an average ≈1.2, and this ratio remained consistently below 2 (Figure 2C). We therefore used the enrichment ratio of 2 as the lower limit to distinguish large granules from small ones. However, when incubating cells expressing NT2RepCT for 16 h in DNP-containing buffer at pH 5, the granules remained unchanged in size and in fluorescence (Figure S6B). The difference in responsiveness to pH implies that NT2RepCT granules are functionally different in the 16 h cells compared to the 6 h ones.

### Low pH Induced NT2RepCT Granules are pH-Reversible and 1,6-Hex Dissolvable

Next, we assessed the material properties of the larger and more protein-enriched granules formed at pH 5 with FRAP. Again, we were restricted to photobleaching the whole intracellular granules because of their small size. We did not observe any fluorescence recovery for them (Figure S6C,D). However, it is still uncertain whether they are irreversible solid aggregates because the partial photobleaching of granules to assess their internal dynamic as a more reliable material properties indicator was unfeasible. We then studied the pH reversibility and 1,6-hex dissolvability of the intracellular granules formed at pH 5. It was noticed that switching the cytosolic pH from 5 back to around 7.5 caused a disappearance of the large granules (Figure 2E) and a large drop in the number of cells containing these large and GFP-enriched granules (GFP enrichment >2) (Figure 2F). Through incubation of the cells again with DNP-containing buffer at pH 5, the large granules formed again (Figure 2E). NT2RepCT could undergo at least two rounds of assembly–disassembly in response to the repeated cycles of pH 5 to 7.5 change (Figure 2F). When cells with large granules formed at pH 5 were treated with 5% 1,6-hex for 2 min directly after the pH 5 buffer was washed away, the addition of 1,6-hex also resulted in the disappearance of most of the granules in the cells (Figure 2E,G). Similar to the pH cycling, granules could also go through at least two rounds of assembly–disassembly by changing the concentration of 1,6-hex (Figure 2E,G). These experiments collectively suggest that the low pH induced formation of large granules is highly reversible.

Both the extensive assembly and dissolution of NT2RepCT upon change in pH and availability of 1,6-hex occurred rapidly and repeatedly on the time scale of few minutes (Figure 2A and Figure S6), a striking difference from yeast stress granules whose prominent formation and deformation require from minutes to hours after pH adjustment,^15−17^ suggesting that NT2RepCT granules are distinct from stress granules and independent of active regulatory processes (e.g., protein synthesis and aggregate degradation) in yeast cells.

### Acidification and LLPS Are Essential for Fibrilization of NT2RepCT *In Vitro*

To examine whether the phase behavior of NT2RepCT in yeast cells under different conditions is correlated with that *in vitro*, a closer study of the properties of purified NT2RepCT was made. All *in vitro* experiments were conducted with the same eGFP variant as that for *in vivo* experiments and in 100 mM sodium phosphate buffer unless otherwise stated.

**Figure 3:**
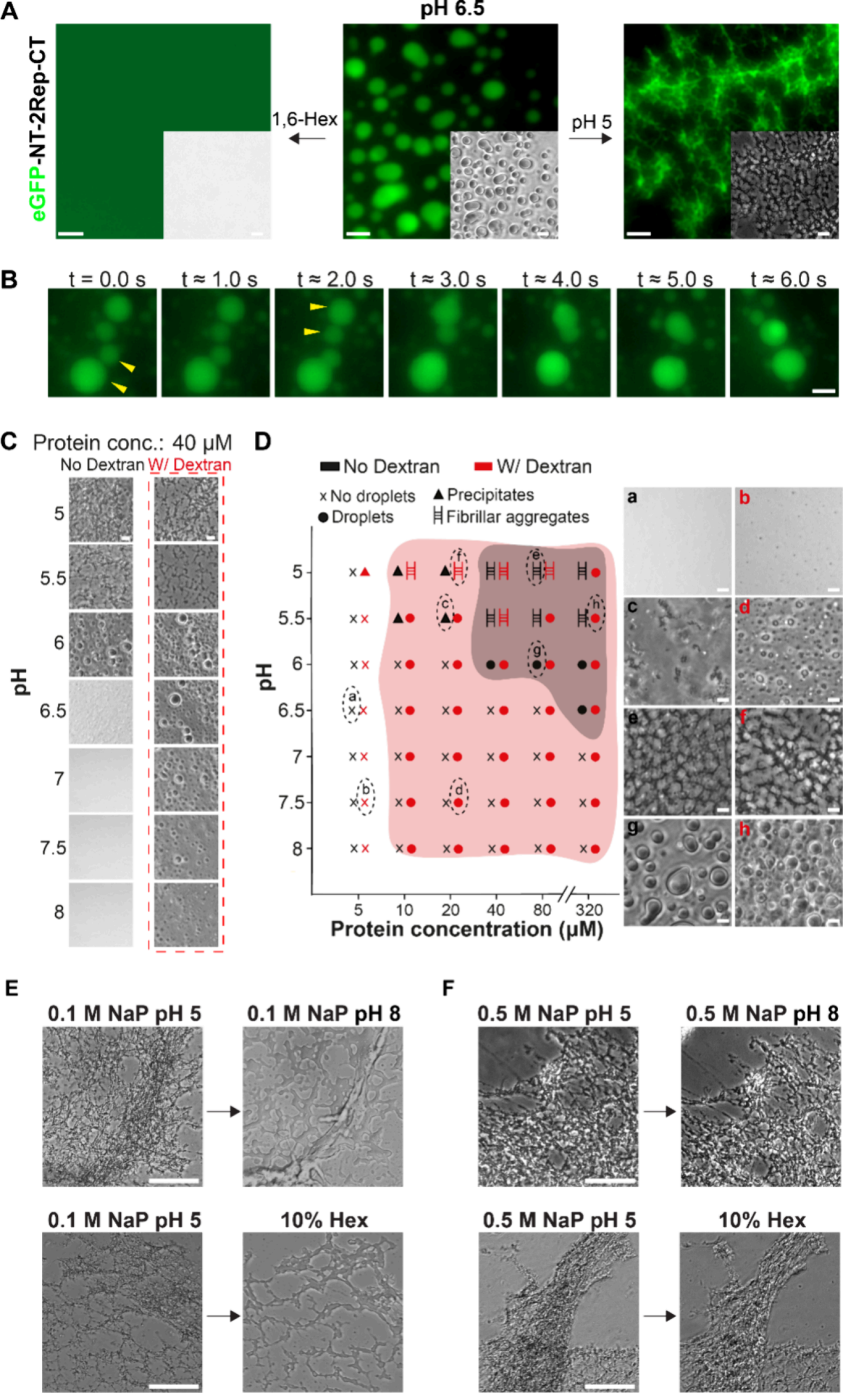
Purified NT2RepCT undergoes LLPS and solidification upon a change in pH and protein concentration. (A) Phase contrast and the corresponding fluorescence microscopy images (green) of NT2RepCT (320 μM) at different conditions. Images show that the protein can form liquid-like droplets in 0.1 M sodium phosphate buffer (pH 6.5) without dextran. The droplets are 1,6-hex dissolvable and can transform into fibrillar-like structures when the pH is lowered to 5. Scale bar in all microscopic images is 5 μm. (B) Dynamic fusion of NT2RepCT droplets (320 μM) at pH 6.5. Scale bar is 5 μm. Yellow arrows indicate fusion event of NT2RepCT droplets. (C) Phase contrast microscopy images of NT2RepCT (40 μM) at 0.1 M sodium phosphate buffer with different pH values with and without dextran. (D) Phase diagram of NT2RepCT with or without dextran. The gray and red areas roughly show the area where NT2RepCT underwent LLPS without and with dextran, respectively. Pictures taken with a light microscope show different structures of NT2RepCT at different regions (marked as a–h) of the phase diagram. Scale bar in all microscopic images is 5 μm. (E) Investigation of pH reversibility and 1,6-hex dissolvability of NT2RepCT fibrils formed at 0.1 M sodium phosphate buffer (pH 5). Scale bar in all microscopic images is 20 μm. (F) Investigation of pH reversibility and 1,6-hex dissolvability of NT2RepCT fibrils formed at 0.5 M sodium phosphate buffer (pH 5). Scale bar in all microscopic images is 20 μm.

We studied the *in vitro* phase behavior of the protein in the same pH range as that in living cells as a function of protein concentration. At pH above 7, no LLPS was observed. At pH 6.5, phase separation occurred only at the protein concentration of 320 μM (≈20 mg/mL), whereas at lower concentrations, the protein remained soluble (Figure S7). The resulting droplets exhibited typical liquid-like condensate properties such as fusion and growth into bigger droplets (Figure 3A,B). They were also dissolved in 5% 1,6-hex (Figure 3A). Decreasing the pH to 6 led to the formation of liquid droplets already at a much lower protein concentration of 40 μM (Figure 3C,D). At pH 5.5 and 5, NT2RepCT no longer underwent LLPS but instead formed aggregates with irregular morphologies at a protein concentration below 40 μM (Figure 3C,D). However, at a higher concentration of >40 μM, which is the critical concentration for LLPS of NT2RepCT at pH above 6, NT2RepCT assembled into networks of extended fibrillar-like structures (Figure 3D and Figure S7).

A major difference between *in vitro* and the cellular environment is that the cytosol is highly crowded, and therefore, experiments were also performed in the presence of a crowding agent. We used 10% dextran as a crowding agent, as it has been demonstrated not to interact with spidroins and this concentration is widely used for mimicking cellular conditions in studies of eukaryotic protein condensates.^50,51^ At pH 8 and in the presence of dextran, LLPS occurred already at a concentration of 10 μM, whereas without dextran at this pH, LLPS was not achieved even at the highest protein concentration tested (320 μM). At pH 5 and with dextran, fibrillation occurred already at 10 μM protein, whereas 40 μM was required without dextran (Figure 3D and Figure S7). However, when a higher protein concentration was used (320 μM) under the same conditions, droplets formed instead of fibrils (Figure 3D and Figure S5). All condensates formed in the presence of dextran showed a characteristic droplet fusion and could be dissolved by 1,6-hex. Overall, we found that dextran effectively lowered the protein concentration required for both LLPS and fibrillation and did not significantly affect the LLPS trend and the low-pH induced liquid-to-solid transition, except for the observation that high protein concentration (e.g., 320 μM) led to liquid-like droplets instead of fibrils.

A surprising finding was that the fibrillar structures of NT2RepCT induced at pH 5 could be solubilized by diluting with an equal volume of buffer at pH 8 or by 10% 1,6-hex (Figure 3E). This occurred in the same way with or without dextran. However, if the concentration of the phosphate buffer at pH 5 was increased from 100 to 500 mM, the fibrils became insoluble in pH 8 or 10% 1,6-hex treatment (Figure 3F). Thus, the solubility of fibrils depends on the concentration of phosphate and not only its presence as previously suggested.^12,13,41^

### Truncation Variants of NT2RepCT Exhibit Decreased Condensation Propensity in Yeast Cells

**Figure 4:**
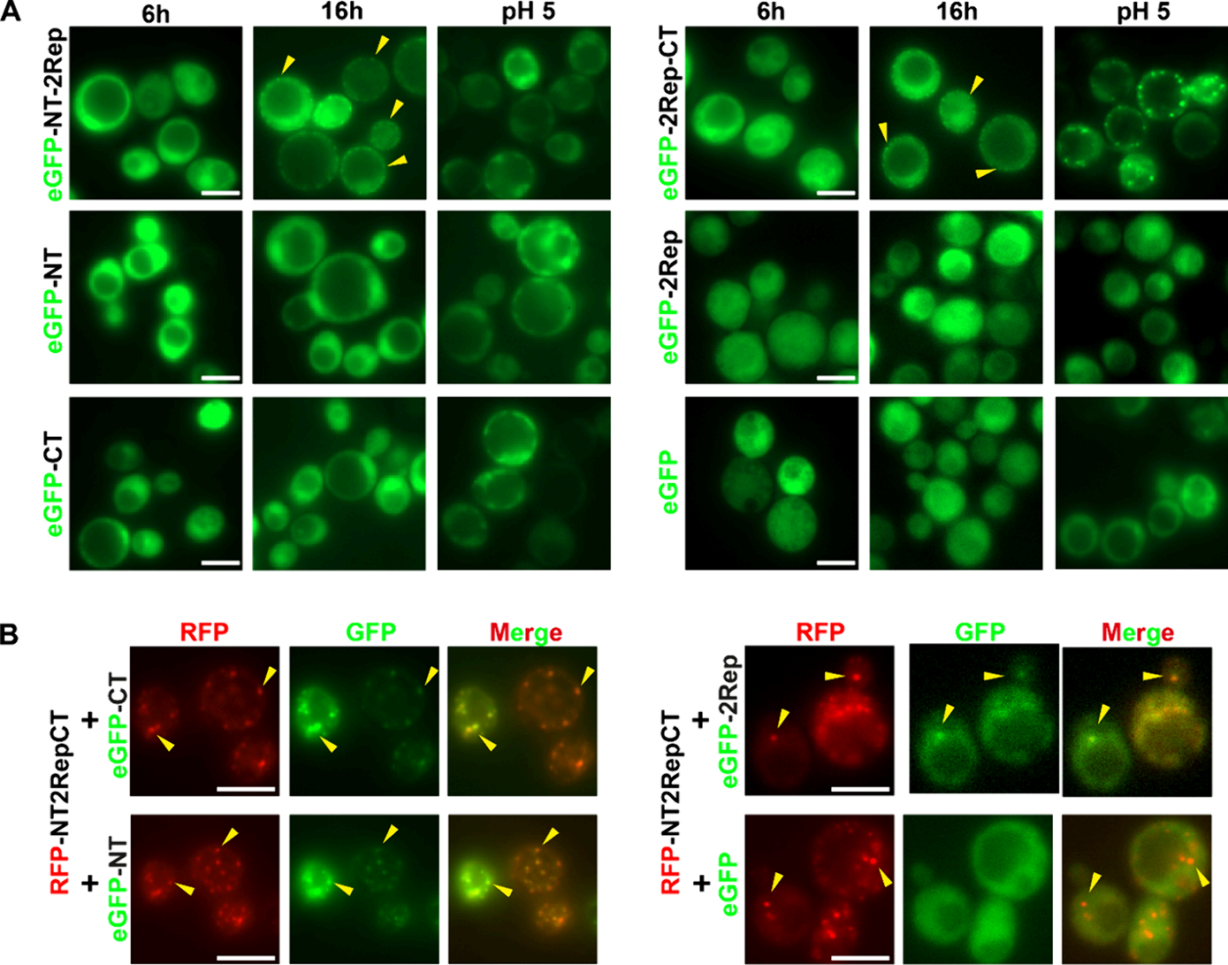
Truncation variants of NT2RepCT exhibit decreased condensation propensity and can colocalize with NT2RepCT in yeast cells. (A) Fluorescence microscopy images of yeast cells expressing different variants for 6 and 16 h and incubated at DNP-containing buffer with pH of 5, respectively. Images were not taken with identical exposure times and fluorescence intensity for clear identification; thus, fluorescence intensities are not comparable. (B) Fluorescence microscopy images of yeast cells coexpressing RFP-NT2RepCT with eGFP-NT, eGFP-CT, eGFP-2Rep, and eGFP, respectively, after adjusting cytosolic pH to pH 5. Yellow arrows indicate intracellular granules within cells. Scale bar in all images is 5 μm.

To further obtain data for the comparison of LLPS *in vivo* and *in vitro*, we studied structural variants of NT2RepCT that are expected to show differences in their LLPS behavior. The variants were the truncated forms: NT2Rep, NT, CT, 2RepCT, and 2Rep. All truncation variants were tagged with eGFP at their N-terminals and expressed in yeast cells under the same conditions as NT2RepCT (Figure S1). The individual domains, i.e., NT, 2Rep, and CT, did not form cytoplasmic granules. They only exhibited diffusive fluorescence in yeast cells in the same way as did eGFP (Figure 4A). NT2Rep and 2RepCT, lacking the CT and NT, respectively, were able to form small granules only after 16 h of expression, a longer expression time compared to NT2RepCT (Figure 4A). These granules were similar in appearance to those formed by NT2RepCT in the early hour postinduction (Figure 4A). Therefore, the 2Rep domain likely needs at least either the NT or CT domain to form intracellular granules, although having only one of the terminal domains leads to LLPS to a much lesser degree than full-domain NT2RepCT.

Next, we studied the effect of pH on truncation variants by adding DNP-containing buffers with a pH range from 8 to 5 to cells. All truncation variants except 2RepCT exhibited a diminished response at pH 5 compared to NT2RepCT (Figure 4A). More specifically, at pH 5, only 2RepCT was able to assemble into larger and more GFP-enriched granules, which were morphologically similar to the NT2RepCT granules formed at the same condition (Figures 2A and 4A). NT2Rep, NT, and CT formed a few big fluorescent puncta in a few cells in a clearly different manner than NT2RepCT, whereas 2Rep remained highly soluble regardless of the expression time (Figure 4A). Comparing the intracellular behaviors of different truncation variants in yeast cells to that of NT2RepCT, the CT domain is believed to be critically important and play a synergistic role with the 2Rep domain for the low pH triggered progressive assembly of NT2RepCT. The accentuated role of CT is in an agreement with many studies demonstrating that the unfolding of the CT domain triggered by low pH initiates the assembly of native spidroins into silk fibers.^36−40^

### Some Truncated Variants Colocalize with NT2RepCT in Yeast Cells

To further illustrate how these individual domains participate in the low-pH induced assembly of NT2RepCT, we coexpressed full-domain RFP-NT2RepCT with the truncated versions eGFP-NT, eGFP-CT, eGFP-2Rep, and eGFP. The coexpression did not significantly affect the behavior of the proteins. Each protein behaved similarly as they were expressed individually in cells (Figure 4A). However, when lowering the intracellular pH to 5, both eGFP-CT and eGFP-NT, which are incapable of condensation by themselves, formed larger, more GFP-enriched and widely distributed granules that colocalized with the granules formed by RFP-NT2RepCT (Figure 4B). We also noticed that eGFP-2Rep was driven to form bigger granules but with lesser colocalization with RFP-NT2RepCT than in the case of eGFP-CT and eGFP-NT (Figure 4B). eGFP remained diffusive in the cytoplasm and did not colocalize with the granules of RFP-NT2RepCT (Figure 4B). The sequestration specificity of NT2RepCT is likely from the specific interactions between NT2RepCT and single-domain variants, reflecting distinct interacting functions of each domain in the NT2RepCT assembly at pH 5.^36−38^

### *In Vitro* LLPS and pH Responsiveness of Truncation Variants Are Similar to Those *In Vivo*

**Figure 5:**
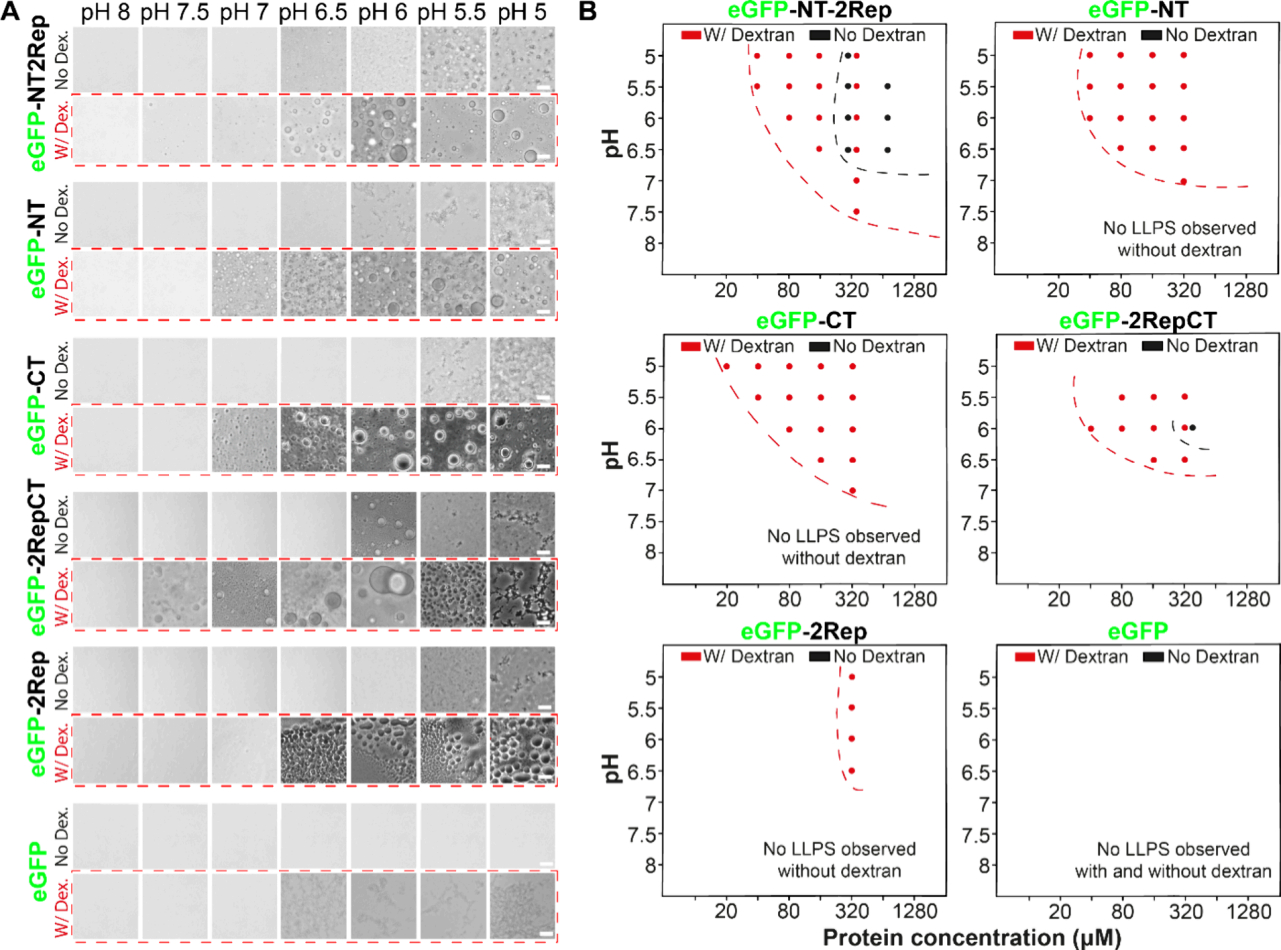
Truncation variants of NT2RepCT can only phase separate under conditions more stringent than NT2RepCT in vitro. (A) Microscopy images of different truncation variants of NT2RepCT at 0.1 M sodium phosphate buffers with different pH values in the presence or absence of dextran. Scale bar in all images is 10 μm. (B) Comparison of the phase diagram of different truncation variants of NT2RepCT with or without dextran. Red dots represent the conditions at which the proteins can undergo LLPS in the presence of dextran, whereas black dots represent the occurrence of LLPS in the absence of dextran. The dash lines roughly represent the area where the LLPS can occur.

We next investigated the behavior of purified truncation variants *in vitro* for a comparison to the above *in vivo* results. In the absence of dextran, only NT2Rep and 2RepCT could undergo LLPS at pH above 6, but both variants required much higher protein concentration (>320 μM) compared to NT2RepCT that already showed LLPS at 40 μM (Figure 5A,B and Figures S8 and S11). Other variants at the same condition were mostly soluble and only assembled into loose aggregates at high protein concentrations (Figure 5A,B and Figures S7–S13). The finding that both NT2Rep and 2RepCT have a higher propensity to undergo LLPS was consistent with the observations *in vivo*.

With the addition of dextran, all truncation variants could undergo LLPS at pH above pH 6 (Figure 5A,B and Figures S7–S13). However, they showed different propensities for LLPS. More specifically, all variants except eGFP-2Rep underwent LLPS at pH above 6 with a minimal protein concentration of around 40 μM (Figure 5B and Figures S7–S13). eGFP-2Rep was highly prone to aggregate and only formed liquid droplets at a high protein concentration of 320 μM. Overall, dextran is likely to promote LLPS by decreasing the minimal saturation concentration of LLPS for all variants.

When the pH of the buffer without dextran was dropped to 5.5 or 5, only 2RepCT was able to form networks of fibrils at all tested concentrations (Figure 5A and Figures S7–S13). However, the fibril networks of 2RepCT were less extensive than the corresponding ones of NT2RepCT (Figures S7 and S11). In the presence of dextran, we noticed that all variants lacking CT domain underwent LLPS at pH 5.5 or 5 but not fibrillation, as observed for 2RepCT and NT2RepCT (Figure 5A and Figures S7–S13). That only 2RepCT and NT2RepCT can assemble into a network of fibrils represents a parallel to our intracellular characterization in yeast cells in which only 2RepCT and NT2RepCT could form large granules in response to lower pH (Figure 5A and Figures S7–S13).

## Discussion

Our result showed that minispidroin NT2RepCT undergoes an LLPS-like process to form liquid condensate-like structures during overexpression in yeast cells. Three lines of evidence are summarized here. First, NT2RepCT was initially present in a soluble form; however, when protein concentration increased, it assembled into condensed granular structures. These granules can be disassembled by 1,6-hex and were distinct from aggregated proteins that trigger UPR in yeast cells, indicating that they probably acquire liquid-like material properties, and the molecules within granules are kept together by weak interactions. Second, these granules were still pH-responsive and further assembled into bigger condensed granules at lower cytosolic pH with their soluble counterpart, in contrast to the 1,6-hex resistant variant that was inert to pH change and was less diffusive. Third, the purified NT2RepCT at 40 μM underwent LLPS *in vitro* at near-physiological pH and intermediate ion strength. In the presence of a crowding agent to mimic cellular conditions, LLPS occurred already at 10 μM, and the droplets can also be dissolved by 1,6-hex.

Although the phase behaviors of NT2RepCT are correlated between *in vivo* and *in vitro* as expected, we still did a detailed characterization of NT2RepCT both *in vivo* and *in vitro* to understand how the intracellular environment affects protein condensation in comparison to systems with purified proteins. This understanding is important for evaluating whether *in vivo* analysis of newly designed material proteins can be leveraged to assess their LLPS propensity and material properties *in vitro*. As a first step, it must be critically evaluated if the intracellular granules truly have liquid condensate properties and assembled through LLPS. According to the general thermodynamic properties of condensates, we expected intracellular NT2RepCT granules to grow larger by fusion or Ostwald ripening,^52,53^ as we clearly observed *in vitro*. We previously found that intracellular droplets of NT2RepCT showed the expected increase in size when produced in *E. coli*.^33^ However, our present study showed that NT2RepCT granules in yeast cells did not noticeably grow beyond a certain size. We also did not observe any fusion events for the granules.

The granules did show a marked growth in size when decreasing the intracellular pH. Decreasing pH is known to induce dimerization of the NT domain and a conformational switching in the CT domain that in turn is connected to a structural change in the 2Rep region.^36,38,41^ These changes together lead to an increased number of intermolecular interactions. The large granules of NT2RepCT were easily dissolved by increasing the pH or adding 1,6-hex. The growth and dissolution could even be repeated for several cycles in the same cells, indicating reversible forming interactions. Although reversibility is an expected property for liquid condensates, it was previously described that for pure NT2RepCT proteins *in vitro*, the corresponding pH switch led to fibril networks and insoluble aggregates.^13,37^ This contradiction can be attributed to the environmental difference between living cells and the *in vitro* conditions. Upon closer examination, we found that if a lower phosphate concentration—0.1M instead of the 0.5 M used in the previous works^13,37^—was used and a crowding agent was present, the purified protein at high concentration of 320 μM formed large reversible droplets instead of irreversible fibrils (Figure S7). That is, the behavior of purified NT2RepCT did resemble more closely the intracellular one when conditions were adjusted to match more closely those of the cell.

The *in vivo* and *in vitro* behaviors of the different truncated versions of NT2RepCT also showed strong similarities. First, only the NT2Rep and 2RepCT variants formed small granules in yeast cells at regular growth conditions, and they both require a longer expression time than NT2RepCT did. NT2Rep and 2RepCT also formed liquid droplets in *vitro* with and without dextran but to a lesser degree than NT2RepCT. Second, 2RepCT did and NT2Rep did not assemble into bigger granules in the low pH of the cytoplasm. Similarly, 2RepCT did and NT2Rep did not form networks of elongated fibrils *in vitro* at pH 5 as NT2RepCT did. Third, the other truncation variants required more stringent conditions to form liquid droplets *in vitro*, such as higher protein concentration, lower pH, and the addition of dextran. Correspondingly, the other truncation variants did also form puncta at low pH in the cytoplasm but with a clearly irregular appearance different from NT2RepCT and 2RepCT. Both *in vitro* and intracellular characterizations identify the combination of 2Rep and CT as the most critical parts of the protein assembly. The NT domain seems to play a nonessential but still enhancing role. This finding is in agreement with other studies on the roles of the different silk protein domains.^13,36,38−40^

The combined results show consistently that *in vitro* LLPS and *in vivo* behavior follow the same pattern, leading us to conclude that intracellular NT2RepCT granule formation is through the process of LLPS and that the granules most likely are condensates. We have found previously that NT2RepCT condensates can grow in size in *E. coli* but not in yeast cells, and therefore, we can draw some general conclusions for the intracellular behavior of NT2RepCT in different cellular systems. Condensate growth seems to be much more restricted in yeast compared with *E. coli* and *in vitro*. There could be several reasons for this. One possibility is the physical–chemical conditions (e.g., ionic strength, salt type, and crowding) of the yeast cytosol, which are unknown and different from *in vitro* conditions and in *E. coli* cells, restrict the formation and diffusion of condensates. It has been pointed out that some heterotypic interactions in the crowded milieu of cells might inhibit intermolecular interactions between condensing molecules and thereby inhibit LLPS tendency.^54^ An alternative explanation is drawn from the NT2RepCT behavior in *E. coli* cells. It was suggested that nucleoid occlusion during *E. coli* cell division resulting in the accumulation of proteins at cellular poles facilitates the initial condensation of self-assembling NT2RepCT.^53,55,56^ The subsequent transition of polar assemblies into half-cell sized liquid-like condensates is likely driven by Ostwald ripening through which condensates (mainly with liquid-like properties) grow bigger by sequestering molecules to minimize the interfacial energy in the cellular system.^41,52,57^ Because of the lack of active processes that can facilitate the accumulation of proteins in the yeast’s cytosol, the coalescence or ripening of NT2RepCT granules leading to bigger granules can be restricted. This assumption is supported by a study showing that resilin-like proteins can undergo condensate fusion in *E. coli* cells but not in mammalian cells in which nucleoid occlusion-like processes are absent,^17^ indicating a difference in environmental conditions of cytoplasm and mechanisms that regulate protein diffusion between prokaryotic and eukaryotic cells. Other reports also indicate that engineering synthetic liquid condensates in yeast cells can be more demanding than that in *E. coli*. Obtaining single or several large condensates with liquid-like properties in yeast cells might require more multivalent interactions and/or higher interaction affinity of interacting domains.^42−44^ One study showed that high interaction affinities (dissociation constant *K*_*d*_ ranging from 10^–11^ to 10^–6^ M) between two interacting domains respectively placed in a tetrameric and dimeric system were used to drive the formation of single and dynamic condensate in yeast cells.^44^ In another example, dimerizable elements were added to an RGG domain (an IDR region of LAF-1 protein) or two RGG domains were linked in tandem to form small droplets that later fused and grew into bigger condensates in yeast cells, whereas a single RGG hardly did so.^58,59^ We speculated that NT2RepCT fails to give rise to sufficient multivalent interactions and interaction affinity required for the advanced assembly of NT2RepCT leading to bigger condensates in yeast cell because, under cellular conditions, interactions between NT2RepCT are mainly generated by dimerization of the CT domain and by the weak hydrophobic and hydrogen bonding interactions endowed by the repetitive regions.^60^ Therefore, increasing multivalency and/or affinity of molecular interactions or increasing protein concentration to compensate for the low valency and strength of interactions might facilitate the growth of NT2RepCT assemblies in yeast cells, which was reflected in our experiment where NT2RepCT granules underwent stronger condensation upon decreasing cytosolic pH.

Optimization of native sequences of biomaterial building blocks through biosynthetic engineering for bioinspired material production is difficult, especially when the molecular mechanism governing the protein interactions and material properties is not fully understood. The expanding knowledge of LLPS as an intermediate step toward biomaterial assembly leads to *in vitro* characterization of potential proteins with LLPS capability as a main strategy for screening material-forming proteins. This screening process can be sped up by the use of *E. coli* as a “living test tube” as previously shown.^41^ However, it is not possible to artificially adjust the intracellular pH of *E. coli* without disrupting the cell wall integrity, making it an nonideal platform to characterize material-forming proteins that undergo pH-regulated phase transitions, e.g., spidroins and squid beak proteins.^7,13^ In addition, although *E. coli* as a host usually results in a high production yield of many material proteins, eukaryotic systems are better suited for the production of complex structural proteins. Therefore, yeast cell emerges as a well-suited system to study behavioral changes of material proteins under different cellular pH values owing to the ease of manually changing cytosolic pH and its compatibility with many complex proteins. This is crucial for finding, for example, spidroin variants able to form fibers in the biomimetic spinning process. Our present study suggests it is feasible to assess *in vitro* functionalities of material building blocks of spidroins based on the intracellular behaviors and properties of the engineered synthetic condensates characterized in living yeast cells. Combining the intracellular characterization of proteins in *E. coli* and yeast cells as “condensate test tubes” would give complementary and more rigorous characterizations for the purpose of identifying potential protein candidates for biomaterials, understanding the phase transitions in biomaterial assembly, and selecting optimal production host.

However, the combination of a wide range of behaviors of structural proteins and the unknown solution and crowding effects in cells can make condensate properties difficult to understand.^61−64^ In this regard, the yeast cell is extremely complex. The crowded nature of the cytoplasm could be assumed to drive toward condensation, and indeed, some effects were consistent with crowding *in vitro*. However, others such as the relative difficulty in which condensates formed seemed to be affected in an opposite way compared to crowding. Competing nonspecific interactions could explain the effect. The multiple ways in which we compared *in vivo* and *in vitro* data in this study form a coherent understanding. Although *a priori* prediction of *in vitro* condensate properties based on *in vivo* protein data is complex, specific trends did clearly emerge. This work builds the foundation for predicting *in vitro* phase behavior from protein characterization in yeast cells and provides general insights into engineering synthetic condensates in different cellular systems.

## Materials and Methods

### Plasmid Cloning

The plasmids containing a DNA sequence encoding eGFP-NT2RepCT (flanked with a 6xHisTag at N terminal) protein and RFP (mCherry) were obtained from a commercial supplier (Genscript). DNA sequences of all truncation variants were derived from the DNA sequence of eGFP-NT2RepCT by PCR (Thermo Fisher Scientific). All DNA sequences were cloned into a pET-28a vector (which was genetically modified to have two *Bsa*I restriction enzyme recognition sites in its multiple cloning sites) using golden gate cloning (Thermo Fisher Scientific) for protein expression in *E. coli* or into a yeast-*E. coli* shuttle vector pRS413-Gal, pRS416-Gal, or pRS413-GPD using *Xho*I and *Xba*I restriction enzymes (Thermo Fisher Scientific) for protein expression in *Saccharomyces cerevisiae*. All plasmids were transformed into the chemically competent *E. coli* top 10 cells for plasmid amplification and long-term storage. All plasmids were verified by DNA Sanger sequencing (Eurofins).

### Plasmid Transformation in Yeast

All plasmids with the shuttle vector were transformed into *S. cerevisiae* strain W303 (MATa; *leu2-3,112 trp1-1 can1-100 ura3-1 ade2-1 his3-11,15*) using the standard lithium acetate protocol. Plasmids encoding RFP fusion proteins were transformed into sYR129 yeast strain, which is a derivative of the strain CEN.PK2-1C (MATa; his3Δ1; leu2-3,112; ura3-52; trp1-289; MAL2-8c; SUC2) with a yEGFP (yeast enhanced GFP) expression cassette integrated into its genome. The sYR129 strain was obtained from Prof. Michael K. Jensen (Technical University of Denmark). The synthetic dropout agar medium without histidine (SD-His) and without uracil (SD-Ura) was used to select colonies containing pRS413-Gal/GPD plasmids and pRS416-Gal plasmids, respectively. Co-transformation of these two types of plasmids with different auxotrophic makers into yeast cells was achieved by plating cells on the SD agar medium without histidine and uracil (SD-His-Ura). One liter of SD medium consists of 6.7 g of yeast nitrogen base without amino acids (Sigma-Aldrich), a sufficient amount of Yeast Synthetic Drop-out medium Supplements (Sigma-Aldrich), 20 g of glucose, 20 mM sodium phosphate buffer (pH 6.5), and, when necessary, 20 g of agar.

### Protein Expression in Yeast

Precultures of yeast strain W303 containing specific plasmids were grown overnight (∼16 h) at 30 °C in the SD medium without specific amino acids from a single colony, whereas precultures of yeast strain sYR129 were grown in the mineral medium (MM) supplemented with specific amino acids. Then the next day, cell cultures were diluted 1:20 with the same medium and grown for 4 h. For cells containing pRS-413/416-Gal plasmids, cells were spun down to remove the supernatant and then were washed three times with 100 mM phosphate buffer. To induce protein expression, cells were grown in SC-His/Ura medium containing 2% galactose. Samples were taken every 2 h for microscopy imaging. For cells containing pRS-413-Gpd plasmids, samples were taken for imaging directly 4 h after refreshing the overnight culture.

### Cytosolic pH Adjustment

Yeast cells at different growing stages were collected by mild centrifugation and transferred to 100 mM phosphate buffer of different pH values containing 1 mM 2,4-dinitrophenol (DNP) dissolved in methanol. Control samples were treated equally, but DNP was omitted from the buffer. Prior to every pH adjustment, yeast cells were thoroughly washed three times with 100 mM phosphate buffer before addition of the DNP-containing buffer with desirable pH.

### 1,6-Hex Treatment for Yeast

Yeast cells were harvested as described above and transferred to 5 or 10% (g/L) 1,6-hexanediol (1,6-hex) solution. For the 1,6-hex cycling experiment, yeast cells were thoroughly washed three times with 100 mM phosphate buffer to remove 1,6-hex residues before adding the DNP-containing buffer at specified pH.

### Florescence Microscopy of Yeast

Samples were prepared as described above. Imaging was done with an Axio Observer Z1 (Carl Zeiss, Germany) microscope (100×/1.4 oil objective, 1.6× tube lens, and Andor iXon Ultra 888 camera). The GFP signal was obtained using excitation light at 470 nm while collecting the emitted light of 515–535 nm (10–20% excitation light intensity depending on the time points of cells and 150 ms exposure time). The RFP signal was obtained using excitation light at 590 nm while collecting the emitted light of 610–635 nm, and images were acquired with 90% light intensity and 150 ms exposure time. The *z*-stacks were collected with a spinning disk (on confocal mode) with 200 nm steps and 50% laser power by using a Nikon Ti-E inverted microscope equipped with a Crest Optics X-light V3 spinning disk confocal head. All of the imaging processing and data analysis on microscopic images were done in ImageJ.

### FRAP

Yeast cells suspended in growth media or certain DNP-containing buffers of different pH values were placed on a microscope coverslip. Images were acquired using a Nikon Ti-E inverted microscope equipped with a 60×/1.4 oil objective lens, 1.5× tube lens, and Crest Optics X-light V3 spinning disk confocal head (operated in the widefield mode). The fluorescence signal of eGFP was excited by continuous illumination on samples using 470 nm light from an LDI Laser Diode Illuminator (89 North) at 1% power level with an exposure time of 50 ms while emission light between 485 and 535 nm was collected. A Gataca Systems iLas 2 unit coupled to a 100 mW OBIS LX 405 nm laser was used to generate a circular spot with a diameter of approximately 1 μm for photobleaching. The photobleaching on samples was carried out at a fixed 10% laser power level and with a manually controlled 900 ms exposure time. After photobleaching, the eGFP signal of each sample was acquired using the above setting but with different exposure times ranging from 50 to 500 ms depending on the samples to minimize imaging-induced photobleaching. Processing of the images and calculation of the fluorescence recovery were carried out using the Fiji (2.3.0) software. FRAP analysis was performed using the ImageJ plugins from Jay Unruh at the Stowers Institute for Medical Research (Kansas City, MO).^65^

### Protein Expression in *E. coli*

All plasmids with the modified pET-28 expression vector were transformed into strain BL21AI *E. coli* (Thermo Fisher Scientific). Precultures of *E. coli* cells were grown in LB media containing 50 μg/mL kanamycin overnight at +30 °C at 220 rpm. Next day, the precultures were diluted 1:100 with the same media and incubated in the same conditions until OD600 reached 0.6. Then, the protein expression was induced with 0.2% l-arabinose and 0.5 mM IPTG and carried out at 20 °C for 18 h. Protein expression was verified by sodium dodecyl sulfate–polyacrylamide gel electrophoresis (SDS-PAGE). Cells were harvested by centrifugation (5000 rpm, +4 °C, 10 min). Cell pellets were frozen at −20 °C.

### Protein Purification from *E. coli*

Protein expression for protein eGFP-NT2RepCT and all its deletion variants was carried out in 500 mL of culture volume using the same protocol as described above. Cells were harvested by centrifugation (5000 rpm, +4 °C, 10 min). Lysis buffer (20 mM Tris-HCl, pH 8) containing 1× protease inhibitor (Thermo Fisher Scientific) was used to resuspend the cell pellets, which was then stored at −20 °C. Next day, frozen cells were thawed, and cell lysis was performed using an Emulsiflex cell homogenizer (18,000 psi, +4 °C). The soluble fraction was separated from cell debris after centrifugation at 20,000 rpm at +4 °C for 20 min and loaded onto a 5 mL Ni-NTA column connected to an AKTA pure (GE Healthcare) chromatography system. The target protein was eluted from the column with 20 mM Tris-HCl (pH 8) containing 250 mM imidazole. The eluted protein was dialyzed against 20 mM Tris-HCl pH 8 at +4 °C using a SnakeSkin dialysis membrane (Thermo Fisher Scientific) with a 3.5 kDa molecular-weight cutoff. Protein purity and integrity were verified with SDS-PAGE.

### Phase Diagram of Proteins *In Vitro*

An Axio Z1 inverted optical microscope (Carl Zeiss, Germany) was used to image the protein samples. The microscopy images were acquired using a 40× magnification objective and Axiocam 503 color camera. Each protein sample (stored in 20 mM Tris-Cl pH 8.0) with a different protein concentration was first pipetted on a glass slide and then mixed with an appropriate buffer in a 1:1 volume ratio. Concentrating protein to a certain concentration was conducted using Vivaspin protein concentrator spin columns (Cytiva), and the protein concentration was measured by a Nanodrop Microvolume Spectrophotometer (Thermo Fisher Scientific). To investigate the influence of pH on protein phase behaviors, the following buffers were used: sodium phosphate (from pH 8.0 to 5.0) and sodium acetate (pH 5.5, 5.0). The final concentration of each buffer was 100 or 500 mM. The different sodium phosphate buffers were prepared by mixing calculated ratios of 1 M stock components (disodium hydrogen phosphate, sodium dihydrogen phosphate, and phosphoric acid). Similarly, the sodium acetate buffers were prepared by mixing calculated ratios of 1 M sodium acetate to acetic acid. The correct pH values were confirmed by using a pH meter. If necessary, buffers containing 10% (w/v) of Dextran 500 (Amersham Biosciences) were used to investigate the effect of crowding agent on protein phase behaviors.

## Supporting Information Available

Schematic representation of NT2RepCT and its truncation variants, SDS-PAGE image of all proteins used in the study, fluorescence microscopy images of NT2RepCT at different time points postinduction and under different conditions, FRAP data of eGFP expressed in yeast cells, unfolding protein response of different proteins in yeast cells, time-lapse images of yeast cells expressing NT2RepCT in the presence of DNP-containing buffer with pH 5, FRAP data of large granules of NT2RepCT at pH 5, microscopy images of purified NT2RepCT and its truncation variants at different conditions, and amino acid sequence of all proteins used in the study (PDF)

## Abbreviations

LLPS: liquid–liquid phase separation
eGFP: enhanced green fluorescent protein
NT2RepCT: a chimeric spidroin
NT: the N-terminal domain of NT2RepCT
2Rep: the repetitive region of NT2RepCT
Rep: the repetitive region of native spidroin
RFP: red fluorescent proteins
MBP: maltose binding protein
LipPks: reference misfolded aggregates
Gal1: galactose inducible promoter
FRAP: fluorescence recovery after photobleaching
1,6-hex: 1,6-hexanediol
NaP: sodium phosphate
IDP: intrinsically disordered protein
IDR: intrinsically disordered region
IPTG: isopropyl-β-d-1-thiogalactopyranoside
LB: lysogeny broth
OD600: optical density at 600 nm
SD-His: synthetic dropout media lacking histidine
SD-Ura: synthetic dropout media lacking uracil
SD-His-Ura: synthetic dropout media lacking histidine and uracil
