# Supplementary material for: A Minispidroin Guides the Molecular Design for Cellular Condensation Mechanisms in S. cerevisiae
Source: ACS Synth Biol. 2023 Sep 9;12(10):3050–63. doi: 10.1021/acssynbio.3c00374 (PMC10594646; doi:10.1021/acssynbio.3c00374)
Supplement: Supplementary file 1 — sb3c00374_si_001.pdf [file sb3c00374_si_001.pdf]

Supporting information: A mini-spidroin guides molecular design for cellular condensation mechanisms in *S.cerevisiae*

Jianhui Feng, Bartosz Gabryelczyk, Isabell Tunn, Ekaterina Osmekhina, Markus B. Linder*

Department of Bioproducts and Biosystems, School of Chemical Engineering and Academy of Finland Center of Excellence in Life-Inspired Hybrid Materials (LIBER), Aalto University, Espoo 02150, Finland.

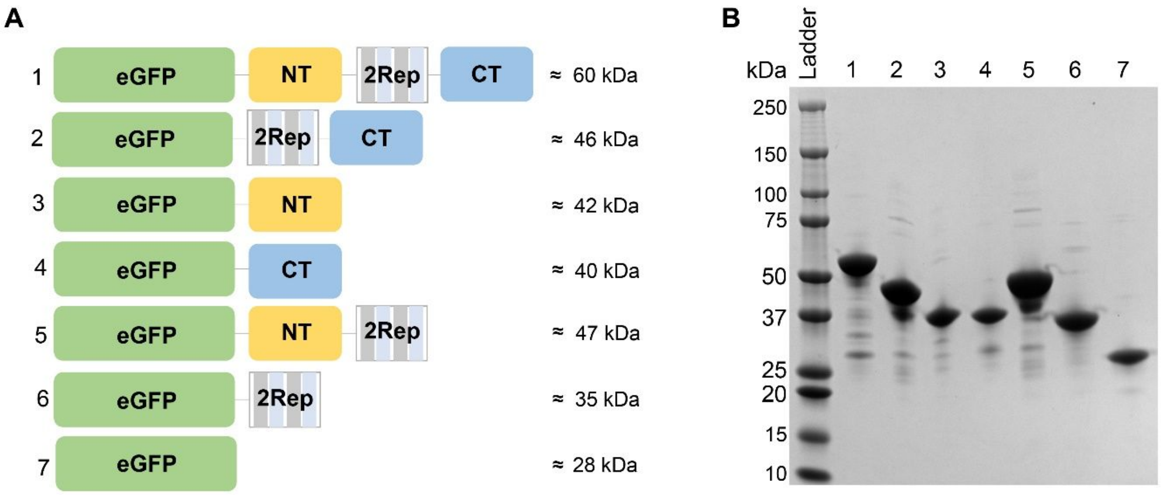

Figure S1: (A) Schematic representation of the proteins and their molecular weight used in this study. (B) Confirmation of purifed 6xHis tagged proteins used in this study. 1: eGFP-NT2RepCT; 2: eGFP-2RepCT; 3: eGFP-NT; 4: eGFP-CT; 5: eGFP-NT2Rep; 6: eGFP-2Rep; 7: eGFP.

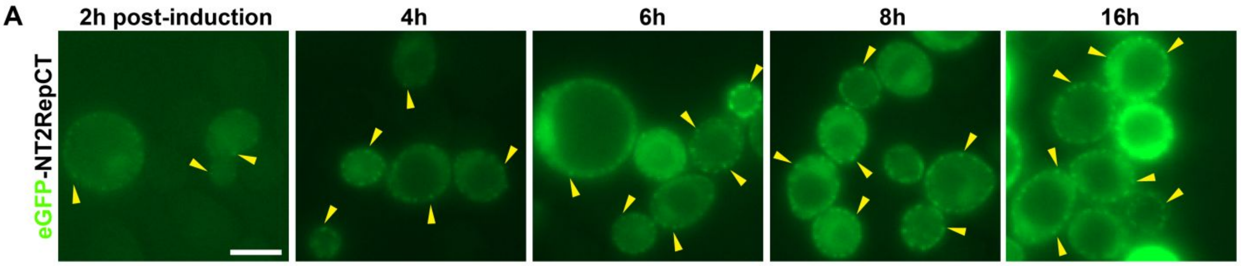

Figure S2: Fluorescence microscopy image of yeast cells expressing NT2RepCT at different time points after induction. Yellow arrows represent the NT2RepCT granules within cells. Scale bar: 5 µm.

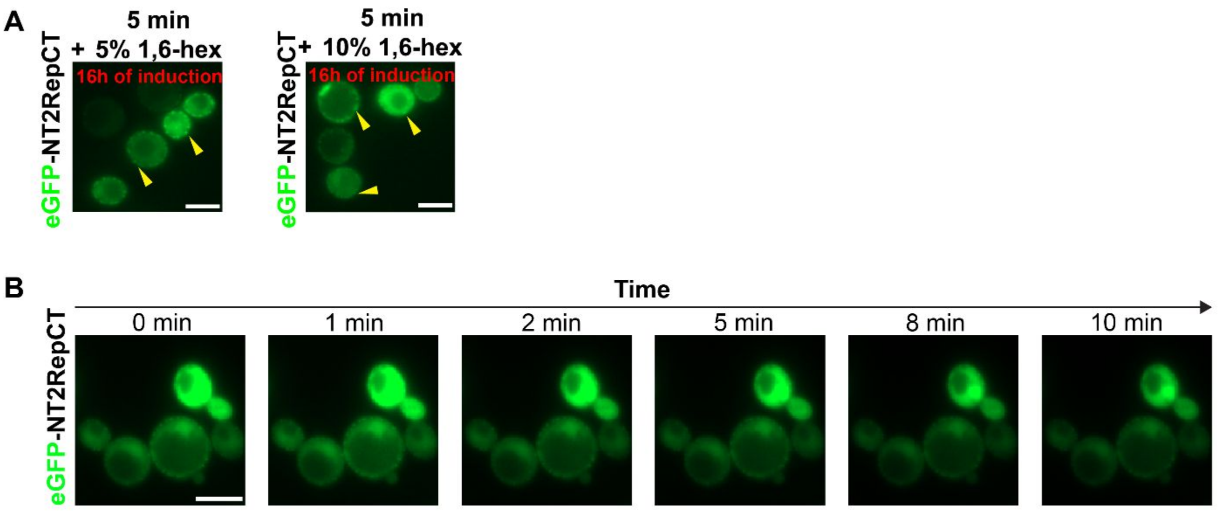

Figure S3: (A) Fluorescence microscopic images of yeast cells expressing NT2RepCT for 16 hours after being treated with 5% (left) and 10% 1,6-hex (right) for 5 minutes. Yellow arrows represent the NT2RepCT granules within cells. (B) Time-lapse fluorescence images of yeast cells expressing NT2RepCT for 6 hours without treatment of 1,6-hex as a control. Scale bar in all microscopic images is 5 µm.

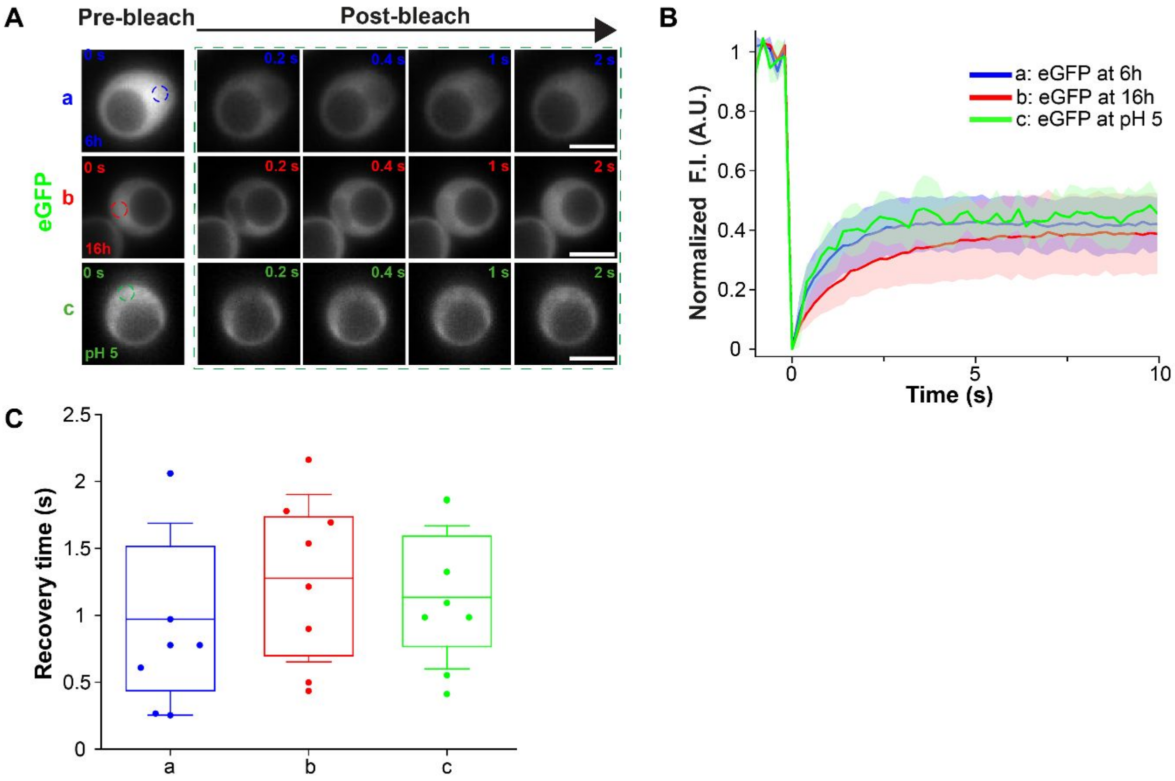

Figure S4: (A)Fluorescence recovery after photobleaching (FRAP) – images of cells expressing eGFP captured at different time points before and after photobleaching. (a) eGFP after 6 hours induction; (b) eGFP after 16 hours induction; (c) eGFP after pH 5 treatment to the cells. Round circles roughly represent the photobleaching area of the cells. Yellow arrows represent the NT2RepCT granules within cells. Scale bar: 5 µm. (B) Recovery of fluorescence of eGFP at different conditions. The bold lines represent the average recovery of fluorescence over time, and the standard deviations of each condition are plotted in the form of a shaded area. (C) The plot of the characteristic time of fluorescence recovery of eGFP at different conditions. Dots represent different replicate samples for measuring (8 replicates for each condition were used for calculation). The mean is shown as a line in the middle of the box, and the box shows lines at 25th and 75th percentiles. Whiskers show standard deviation values.

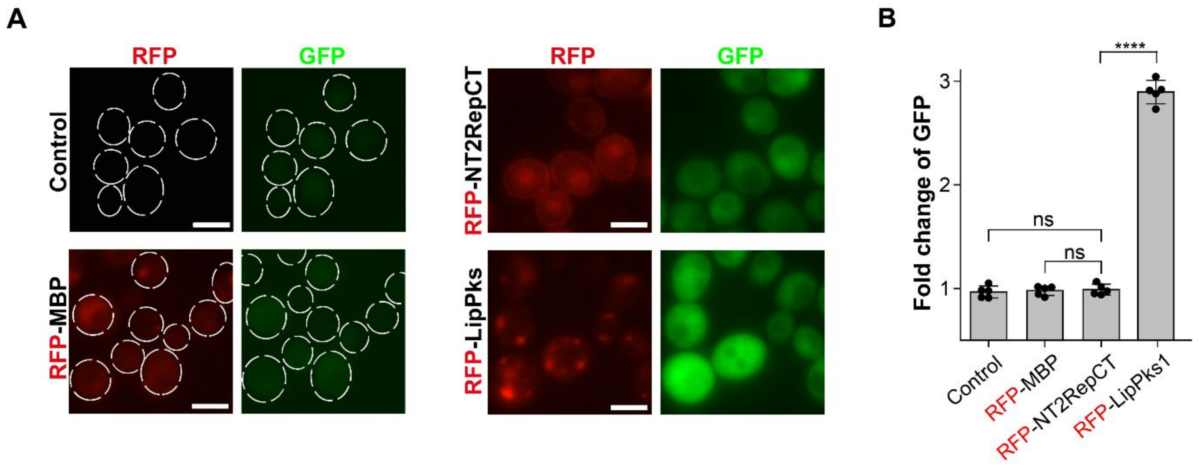

Figure S5: (A) Fluorescence microscopy images of yeast strains (integrated with reporter gene expression cassette controlled by UPR-responsive promoter) expressing RFP-MBP, RFP-LipPks, and RFP-NT2RepCT and the control strain (without heterologous protein). Dash circles represent cell border. (B) Fluorescence measurement of yeast strains grown for 16 hours. For each heterologous protein, the fold change was calculated by normalizing GFP geometric means for each replicate to the GFP geometric mean of all RFP-MBP replicates. Five biological replicates were used for measurement. Bars represent the mean and whiskers show standard deviation values. Asterisks represent statistical significance between conditions indicated by horizontal bar ends: **** = $p < 0.0001$, *** = $p < 0.001$, ** = $p < 0.001$, * = $p < 0.05$, and ns = not significant ($p > 0.05$). Scale bar of all microscopic images, 5 µm.

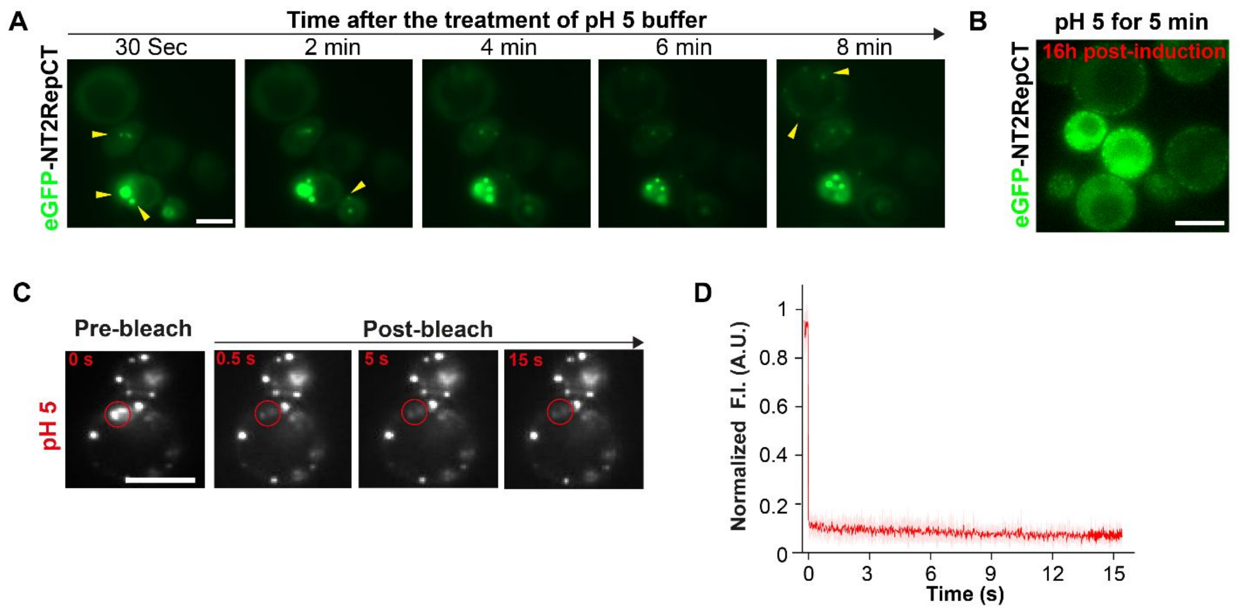

Figure S6: (A) Time lapse images of yeast cells expressing NT2RepCT in the presence of DNP-containing buffer with a pH of 5. Yellow arrows represent the NT2RepCT granules within cells. (B) Fluorescence microscopic images of yeast cells expressing NT2RepCT for 16 hours after being treated with DNP

containing buffer of pH 5 for 5 minutes. (C) Fluorescence recovery after photobleaching (FRAP) – images of cells expressing NT2RepCT captured at different time points before and after photobleaching. Before photobleaching, cells were treated with pH 5 buffer containing DNP to lower cytosolic pH. Red circles depict the bleached area on the cells. (D) Recovery of fluorescence of bigger granules of NT2RepCT at pH 5. The plot shows the average recovery of fluorescence of such granules. The bold lines represent the average recovery of fluorescence over time, and the standard deviations of each condition are plotted in a form of shaded area.

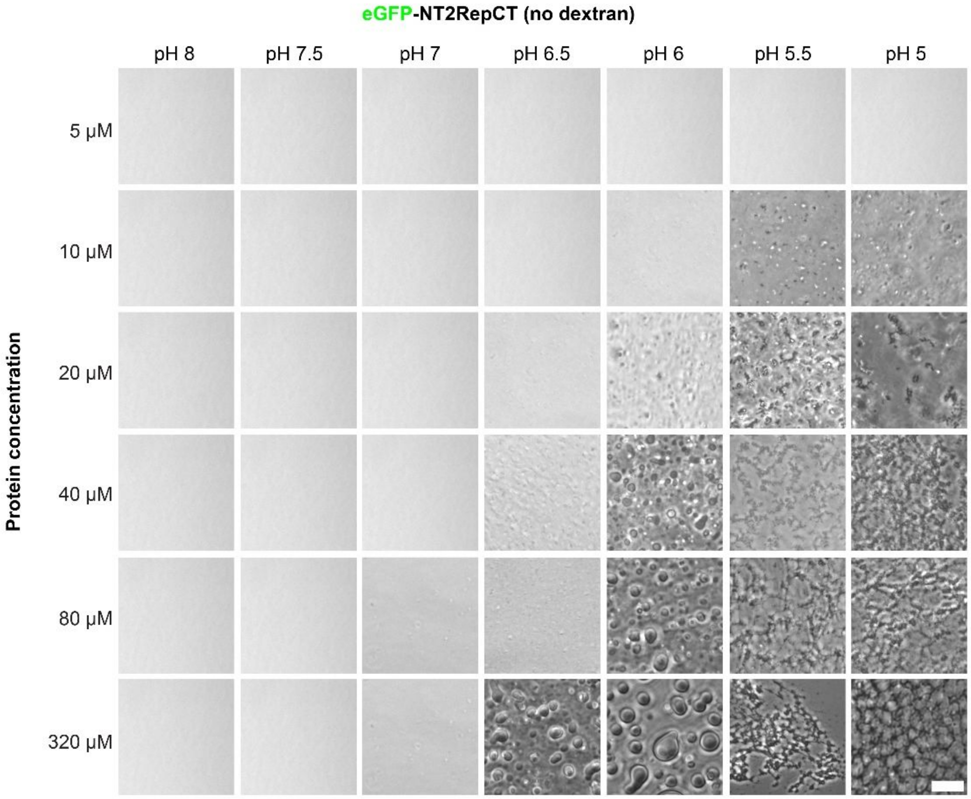

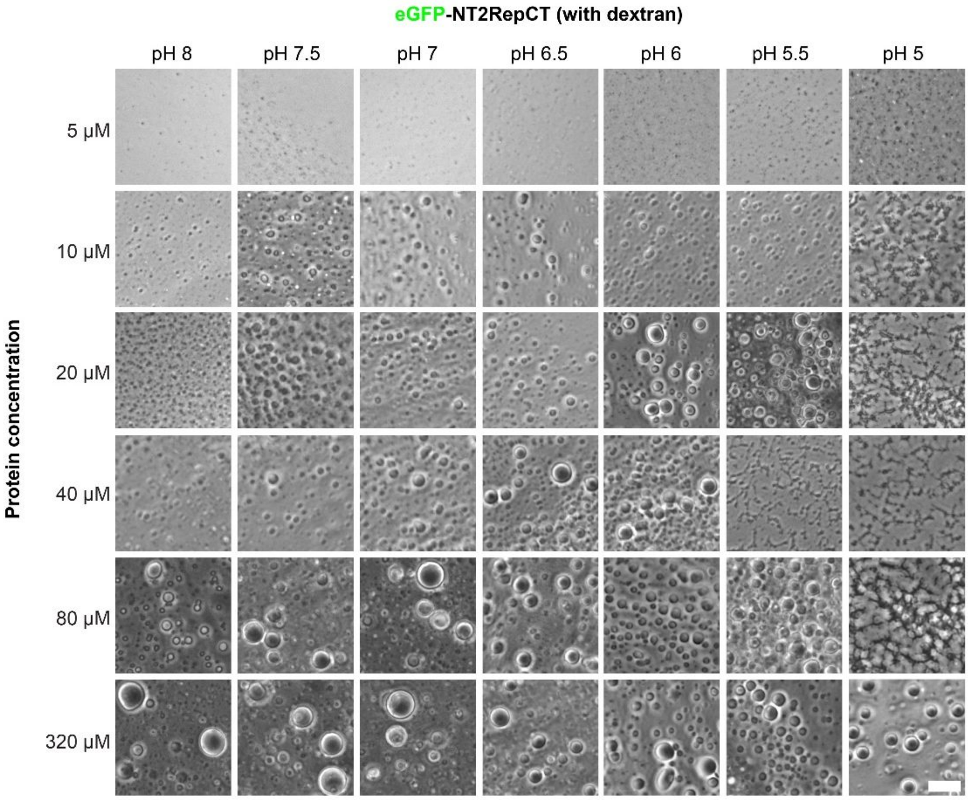

Figure S7: Light microscopy images of NT2RepCT under different conditions *in vitro*. Scale bar, 8 µm.

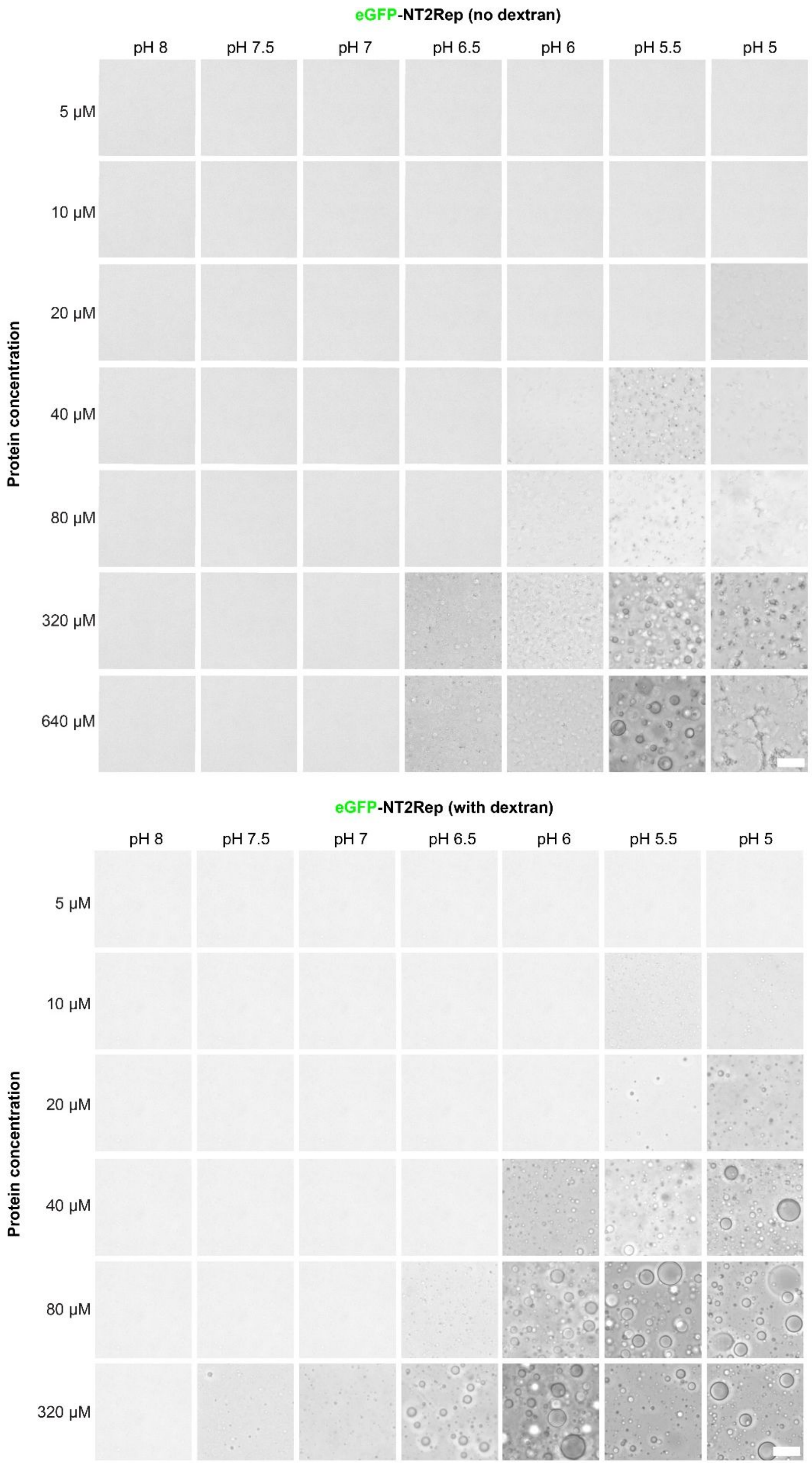

Figure S8: Light microscopy images of eGFP-NT2Rep under different conditions *in vitro*. Scale bar, 8 µm.

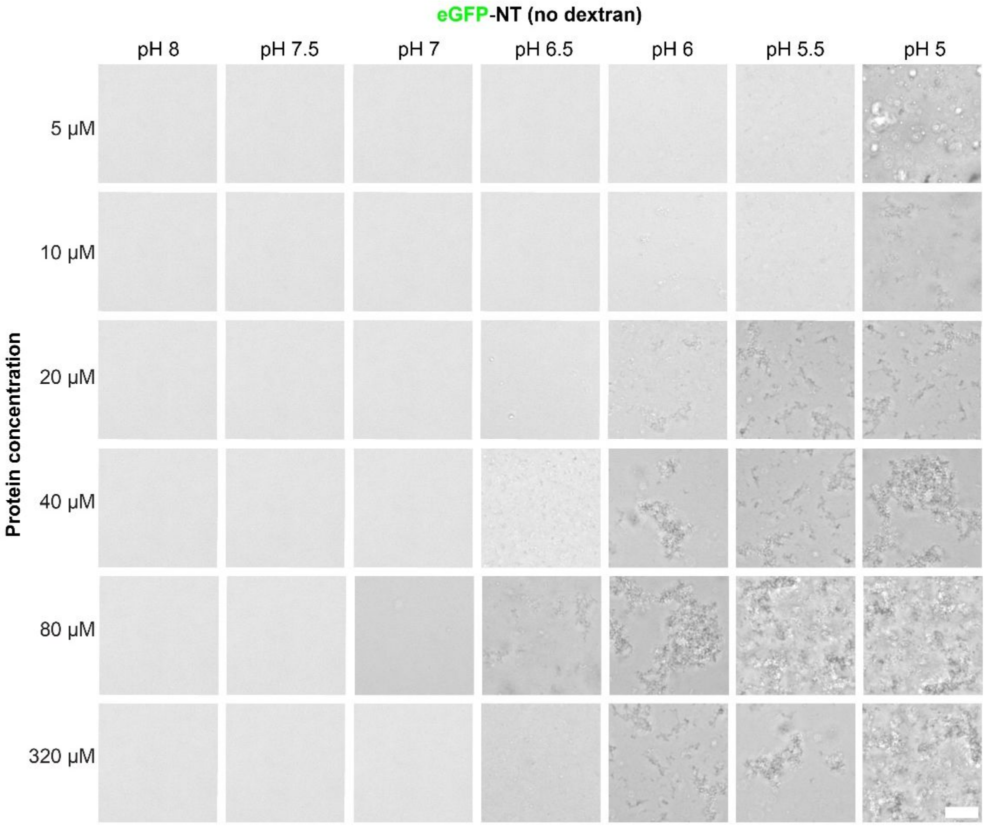
eGFP-NT (no dextran)
pH 8
pH 7.5
pH 7
pH 6.5
pH 6
pH 5.5
pH 5
Protein concentration
5 µM
10 µM
20 µM
40 µM
80 µM
320 µM

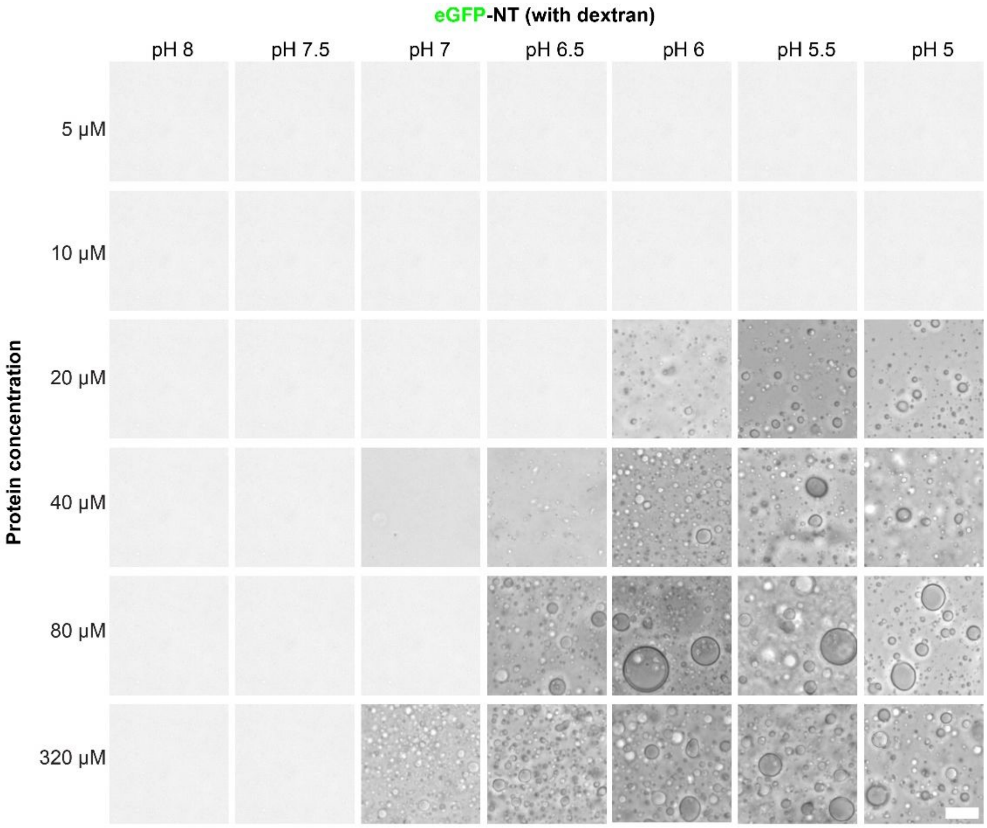

Figure S9: Light microscopy images of eGFP-NT under different conditions *in vitro*. Scale bar, 8 μm.

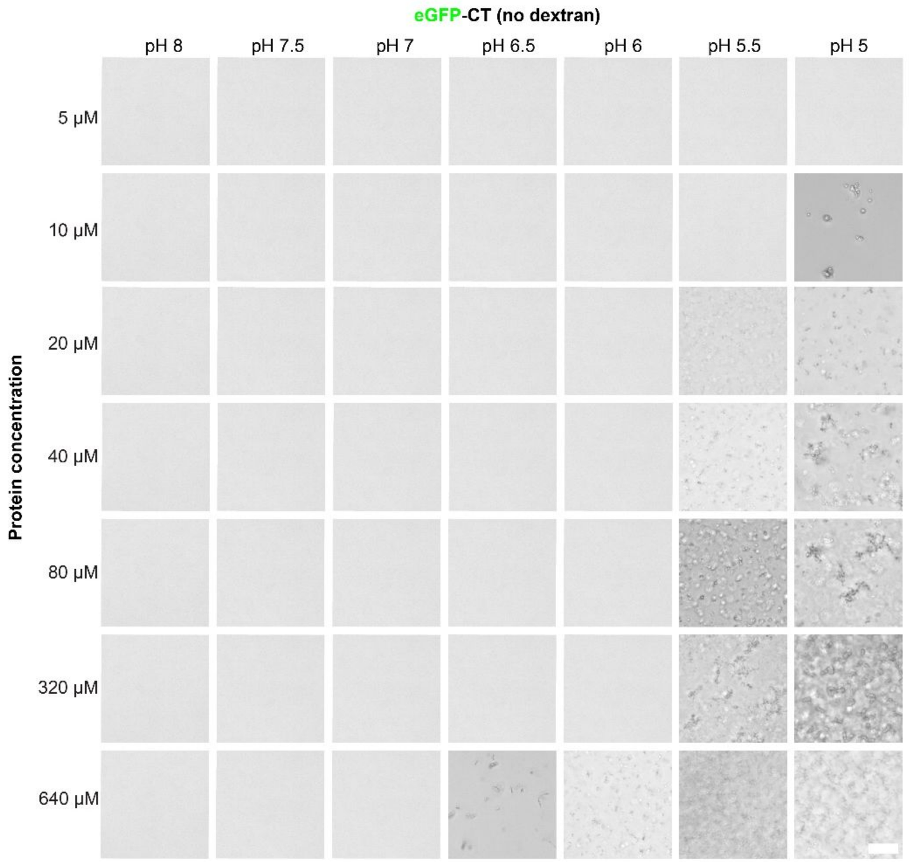
eGFP-CT (no dextran)
pH 8
pH 7.5
pH 7
pH 6.5
pH 6
pH 5.5
pH 5
5 µM
10 µM
20 µM
40 µM
80 µM
320 µM
640 µM
Protein concentration

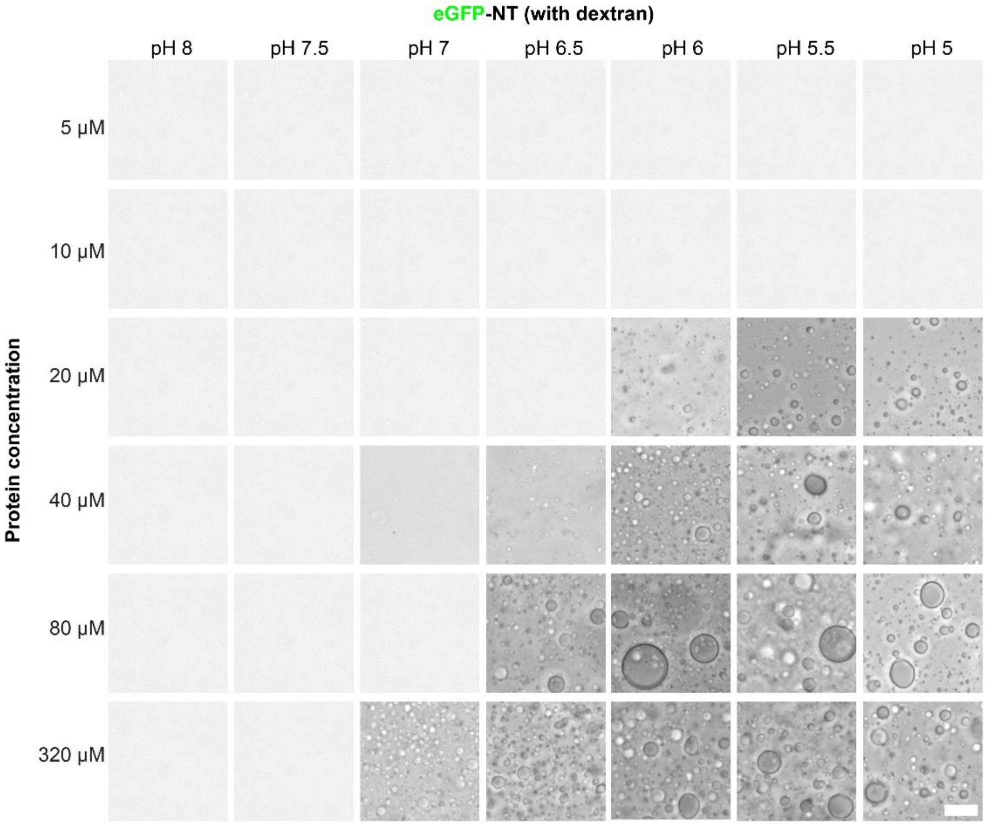
eGFP-NT (with dextran)
pH 8
pH 7.5
pH 7
pH 6.5
pH 6
pH 5.5
pH 5
5 µM
10 µM
20 µM
40 µM
80 µM
320 µM
Protein concentration

Figure S10: Light microscopy images of eGFP-CT under different conditions *in vitro*. Scale bar, 8 μm.

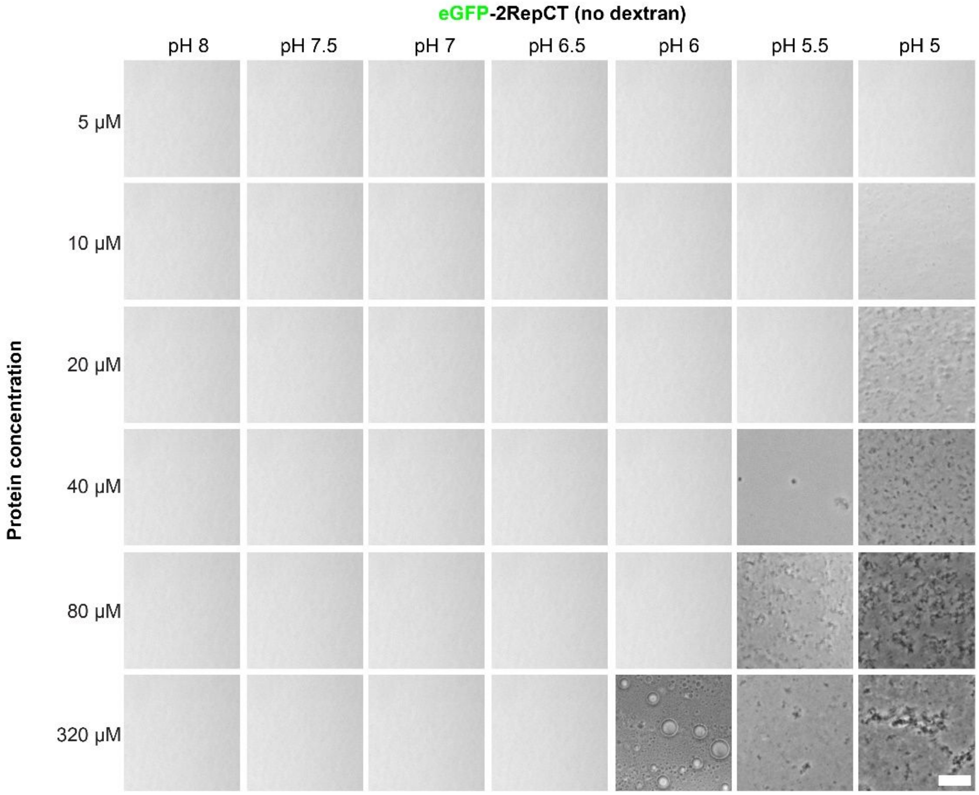

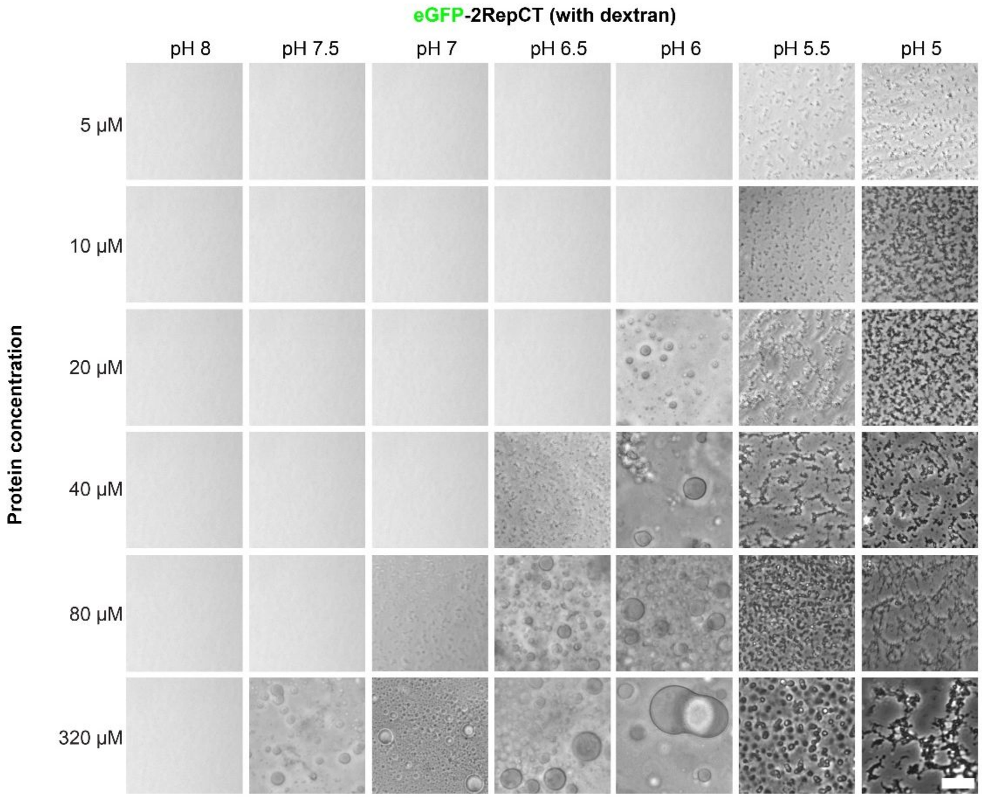

Figure S11: Light microscopy images of eGFP-2RepCT under different conditions *in vitro*. Scale bar, 8 µm.

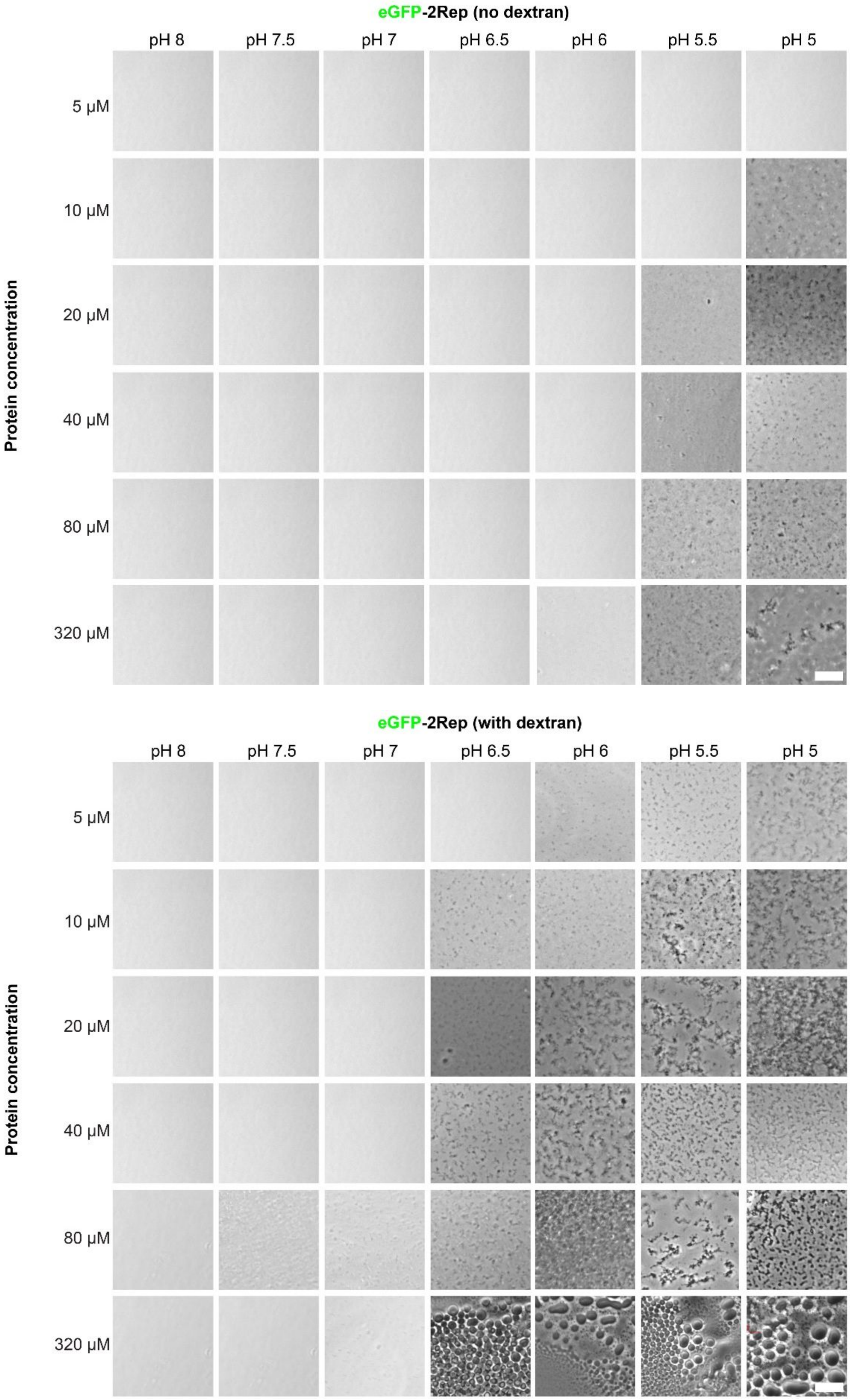

Figure S12: Light microscopy images of eGFP-2Rep under different conditions *in vitro*. Scale bar, 8 µm.

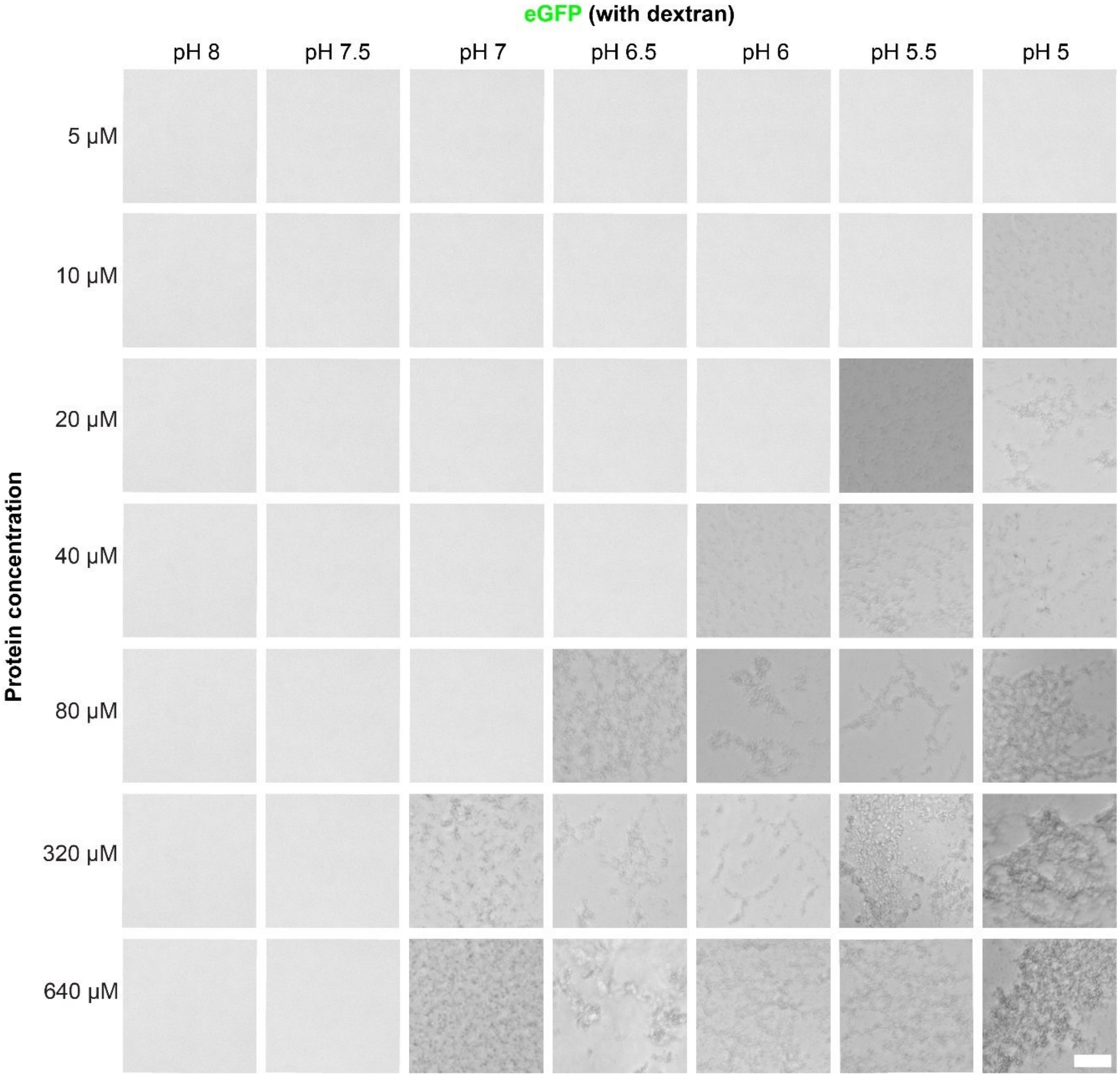

Figure S13: Light microscopy images of eGFP under different conditions in the presence of dextran *in vitro*. Scale bar, 8 μm.

Sequence information:

eGFP-NT2RepCT

MHHHHHHSSGVSKGEELFTGVVPILVELDGDVNGHKFSVSGEGEGDATYGKLTLKFICTTGKLPVPWPTLVTTLTYGVQ
CFSRYPDHMKQHDFFKSAMPEGYVQERTIFFKDDGNYKTRAEVKFEGDTLVNRIELKGIDFKEDGNILGHKLEYNYNSH
NVYIMADKQKNGIKVNFKIRHNIEDGSVQLADHYQQNTPIGDGPVLLPDNHYLSTQSALSKDPNEKRDHMVLLEFVTA
AGITLGMDELYKEFGGGSMSHTTPWTNPGLAENFMNSFMQGLSSMPGFTASQLDDMSTIAQSMVQSIQSLAAQGR
TSPNKLQALNMAFASSMAEIAASEEGGGSLSTKTSSIASAMSNAFLQTTGVVNQPFINEITQLVSMFAQAGMNDVSAG
NSGRGQGGYGQGSGGNAAAAAAAAAAAAAAAAGQGGQGGYGRQSQGAGSAAAAAAAAAAAAAAGSGQGGYGG
QGQGGYGQSGNSVTSGGYGYGTSAAAGAGVAAGSYAGAVNRLSSAEAASRVSSNIAAIASGGASALPSVISNIYSGVV
ASGVSSNEALIQALLELLSALVHVLSSASIGNVSSVGVDSTLNVVQDSVGQYVG

eGFP-NT2Rep

MHHHHHHSSGVSKGEELFTGVVPILVELDGDVNGHKFSVSGEGEGDATYGKLTLKFICTTGKLPVPWPTLVTTLTYGVQ
CFSRYPDHMKQHDFFKSAMPEGYVQERTIFFKDDGNYKTRAEVKFEGDTLVNRIELKGIDFKEDGNILGHKLEYNYNSH
NVYIMADKQKNGIKVNFKIRHNIEDGSVQLADHYQQNTPIGDGPVLLPDNHYLSTQSALSKDPNEKRDHMVLLEFVTA
AGITLGMDELYKEFGGGSMSHTTPWTNPGLAENFMNSFMQGLSSMPGFTASQLDDMSTIAQSMVQSIQSLAAQGR
TSPNKLQALNMAFASSMAEIAASEEGGGSLSTKTSSIASAMSNAFLQTTGVVNQPFINEITQLVSMFAQAGMNDVSAG
NSGRGQGGYGQGSGGNAAAAAAAAAAAAAAAAGQGGQGGYGRQSQGAGSAAAAAAAAAAAAAAGSGQGGYGG
QGQGGYGQSGNS

eGFP-2RepCT

MHHHHHHSSGVSKGEELFTGVVPILVELDGDVNGHKFSVSGEGEGDATYGKLTLKFICTTGKLPVPWPTLVTTLTYGVQ
CFSRYPDHMKQHDFFKSAMPEGYVQERTIFFKDDGNYKTRAEVKFEGDTLVNRIELKGIDFKEDGNILGHKLEYNYNSH
NVYIMADKQKNGIKVNFKIRHNIEDGSVQLADHYQQNTPIGDGPVLLPDNHYLSTQSALSKDPNEKRDHMVLLEFVTA
AGITLGMDELYKEFGGGSGNSGRGQGGYGQGSGGNAAAAAAAAAAAAAAAAGQGGQGGYGRQSQGAGSAAAAAA
AAAAAAAAGSGQGGYGGQGQGGYGQSGNSVTSGGYGYGTSAAAGAGVAAGSYAGAVNRLSSAEAASRVSSNIAAI
ASGGASALPSVISNIYSGVVASGVSSNEALIQALLELLSALVHVLSSASIGNVSSVGVDSTLNVVQDSVGQYVG

eGFP-NT

MHHHHHHSSGVSKGEELFTGVVPILVELDGDVNGHKFSVSGEGEGDATYGKLTLKFICTTGKLPVPWPTLVTTLTYGVQ
CFSRYPDHMKQHDFFKSAMPEGYVQERTIFFKDDGNYKTRAEVKFEGDTLVNRIELKGIDFKEDGNILGHKLEYNYNSH
NVYIMADKQKNGIKVNFKIRHNIEDGSVQLADHYQQNTPIGDGPVLLPDNHYLSTQSALSKDPNEKRDHMVLLEFVTA
AGITLGMDELYKEFGGGSMSHTTPWTNPGLAENFMNSFMQGLSSMPGFTASQLDDMSTIAQSMVQSIQSLAAQGR
TSPNKLQALNMAFASSMAEIAASEEGGGSLSTKTSSIASAMSNAFLQTTGVVNQPFINEITQLVSMFAQAGMNDVSA

eGFP-2Rep

MHHHHHHSSGVSKGEELFTGVVPILVELDGDVNGHKFSVSGEGEGDATYGKLTLKFICTTGKLPVPWPTLVTTLTYGVQ
CFSRYPDHMKQHDFFKSAMPEGYVQERTIFFKDDGNYKTRAEVKFEGDTLVNRIELKGIDFKEDGNILGHKLEYNYNSH
NVYIMADKQKNGIKVNFKIRHNIEDGSVQLADHYQQNTPIGDGPVLLPDNHYLSTQSALSKDPNEKRDHMVLLEFVTA
AGITLGMDELYKEFGGGSGNSGRGQGGYGQGSGGNAAAAAAAAAAAAAAAAGQGGQGGYGRQSQGAGSAAAAAA
AAAAAAAAGSGQGGYGGQGQGGYGQSGNS

eGFP-CT

MHHHHHHSSGVSKGEELFTGVVPILVELDGDVNGHKFSVSGEGEGDATYGKLTLKFICTTGKLPVPWPTLVTTLTYGVQ
CFSRYPDHMKQHDFFKSAMPEGYVQERTIFFKDDGNYKTRAEVKFEGDTLVNRIELKGIDFKEDGNILGHKLEYNYNSH
NVYIMADKQKNGIKVNFKIRHNIEDGSVQLADHYQQNTPIGDGPVLLPDNHYLSTQSALSKDPNEKRDHMVLLEFVTA
AGITLGMDELYKEFGGGSVTSGGYGYGTSAAAGAGVAAGSYAGAVNRLSSAEAASRVSSNIAAIASGGASALPSVISNIY
SGVVASGVSSNEALIQALLELLSALVHVLSSASIGNVSSVGVDSTLNVVQDSVGQYVG
