# Supplementary material for: A Minispidroin Guides the Molecular Design for Cellular Condensation Mechanisms in S. cerevisiae
Source: ACS Synth Biol. 2023 Sep 9;12(10):3050–63. doi: 10.1021/acssynbio.3c00374 (PMC10594646; doi:10.1021/acssynbio.3c00374)

A mini-spidroin guides molecular design for cellular condensation mechanisms in *S.cerevisiae*

Jianhui Feng, Bartosz Gabryelczyk, Isabell Tunn, Ekaterina Osmekhina, Markus B. Linder*

Department of Bioproducts and Biosystems, School of Chemical Engineering and Academy of Finland Center of Excellence in Life-Inspired Hybrid Materials (LIBER), Aalto University, Espoo 02150, Finland.

Table of content:

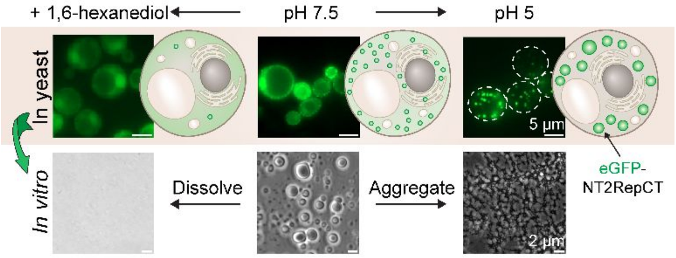

Supplement: Supplementary file 2 — sb3c00374_si_002.pdf [file sb3c00374_si_002.pdf]
